# Integrative Transcriptomic and Network Analysis of Hemocyte Volume Plasticity and Redox Regulation Under Osmotic Stress in *Penaeus monodon*

**DOI:** 10.3390/antiox15010147

**Published:** 2026-01-22

**Authors:** Sheng Huang, Falin Zhou, Qibin Yang, Song Jiang, Jilin Chen, Jie Xiong, Erchao Li, Yundong Li

**Affiliations:** 1 School of Life Sciences, East China Normal University, Shanghai 200241, China; huangshenglcu@163.com; 2 Key Laboratory of South China Sea Fishery Resources Exploitation and Utilization, Ministry of Agriculture and Rural Affairs, South China Sea Fisheries Research Institute, Chinese Academy of Fishery Sciences, Guangzhou 510300, China; zhoufalin0925@163.com (F.Z.); yangqibin1208@163.com (Q.Y.); tojiangsong@163.com (S.J.); 3 Key Laboratory of Efficient Utilization and Processing of Marine Fishery Resources of Hainan Province, Sanya Tropical Fisheries Research Institute, Sanya 572018, China; 4 Hainan Seed Industry Laboratory, Sanya 572024, China; chenjilin1989@fafu.edu.cn (J.C.); xiongjie@caas.cn (J.X.)

**Keywords:** *Penaeus monodon*, hemocytes, salinity adaptation, osmoregulation, ribosomal proteins

## Abstract

Osmotic stress affects ion transport and cell hydration, potentially disrupting redox homeostasis through altered proteostasis and mitochondrial metabolism. However, how immune hemocytes coordinate volume regulation with these stress-linked processes, particularly oxidative stress and antioxidant responses, remains unclear in crustaceans. This study integrated quantitative cytology, RNA sequencing, and network analysis to profile hemocyte volume plasticity in the euryhaline shrimp *Penaeus monodon* across a salinity gradient. Hemocytes were incubated for 24 h in hypoosmotic, isosmotic, and hyperosmotic media, with significant volume shifts observed while maintaining membrane integrity and morphology. The permeability of solutes (urea and sorbitol) suggested that volume adjustment is coupled with solute transport. Transcriptomic analyses identified key salinity-responsive pathways, including oxidative phosphorylation, MAPK signaling, ribosome biogenesis, and antioxidant defense mechanisms, underscoring the activation of redox-regulatory systems under osmotic stress. Weighted gene co-expression network analysis highlighted ribosomal proteins as central hubs in a salinity-responsive module, with qRT-PCR confirming the co-regulation of these hubs alongside representative osmoregulatory and antioxidant genes (*AQP4*, Na^+^/K^+^-*ATPase*, *HSP70*, *CHOP*, and antioxidant enzymes). These findings reveal how hemocyte volume dynamics are coupled to redox regulation, providing a mechanistic framework for understanding osmotic stress–redox coupling in crustacean immune cells.

## 1. Introduction

Salinity variation is one of the key environmental factors influencing the structure and function of marine ecosystems. For invertebrates living in the intertidal zone and nearshore waters, fluctuations in salinity not only determine their geographical distribution and habitat suitability but also directly affect their osmoregulation, energy metabolism, and maintenance of cellular homeostasis [1].

Osmoregulation is the core process by which organisms maintain the balance of ion concentrations and osmotic pressure between the inside and outside of cells under varying salinity conditions, and its efficiency is directly related to the survival, development, and reproductive success of the organisms. Different species have developed a variety of salinity adaptation strategies during long-term evolution, including the regulation of ion pump activity, the accumulation of organic osmolytes, and the dynamic regulation of cell volume. However, in crustaceans, especially hemocytes, which are key units for immune and metabolic regulation, the volume response and molecular regulatory mechanisms to salinity stress are still poorly understood [2].

*P. monodon* is a typical euryhaline crustacean, capable of maintaining vital activities over a wide range of salinities (0–40 and even higher). Its remarkable salinity tolerance makes it an ideal model species for studying the osmoregulatory mechanisms of marine invertebrates [3]. Previous studies have mainly focused on the osmoregulatory functions of gills, hepatopancreas, and muscle tissues in *P. monodon*. For example, Na^+^/K^+^-*ATPase*, Na^+^/H^+^ exchanger (NHE), and chloride channels in the gills are believed to play a core role in ion transport; while in the hepatopancreas, the organism regulates the content of free amino acids (such as glycine, taurine) and trimethylamine oxide to cope with changes in external osmotic pressure. However, as the key executor of systemic osmoregulation and immune response, the volume regulation, cell membrane stability, and ion transport behavior of hemocytes under salinity changes have not been fully studied.

In natural habitats and farming ponds, *P. monodon* routinely experiences salinity fluctuations from near-freshwater to brackish and seawater conditions, typically between approximately 0 ppt and 40 ppt, with occasional exposure to more hypersaline water in shallow, evaporation-driven ponds or during tidal intrusion events. *Penaeus monodon* is a euryhaline penaeid shrimp that experiences substantial osmotic challenges when environmental salinity fluctuates [4]. In this species, hemolymph osmolality varies as a linear function of external medium osmolality (slope ≈ 0.28) and becomes approximately iso-osmotic at ~700–750 mOsm·kg^−1^ (equivalent to ~25–26 ppt), such that conditions below this iso-osmotic point are hypo-osmotic relative to hemolymph (hemolymph remains hyperosmotic to the medium), whereas conditions above this point are hyperosmotic relative to hemolymph (hemolymph becomes hypo-osmotic to the medium). Accordingly, this research uses the terms hypo-/iso-/hyper-osmotic with reference to the iso-osmotic point (osmotic line) rather than “near-optimal” salinity, as these concepts are not necessarily equivalent [5]. Changes in cell volume are one of the most direct manifestations of salinity stress. Most organisms experience rapid, acute cell swelling due to water influx in low-salinity environments because of the reduced external osmotic pressure, while in high-salinity environments, cells acutely contract due to water efflux [6]. However, this classical swelling/shrinkage pattern primarily describes the immediate (minutes–hours) osmotic response. Under prolonged exposure (e.g., 24 h), hemocytes can engage regulatory volume decrease/increase (RVD/RVI), and the apparent mean cell size may also be influenced by shifts in hemocyte sub-population composition. Therefore, cell volumes quantified after 24 h should be interpreted as a regulated steady-state phenotype rather than the initial osmotic swelling/shrinkage phase. However, in isolated hemocytes cultured in vitro, the direction and magnitude of volume change may deviate from this canonical pattern depending on medium ionic composition, solute permeability, and regulatory volume responses (RVD/RVI), underscoring the need for permeability-matched osmolyte controls when interpreting salinity–volume relationships. During this process, cells can regulate ion transport and the activity of water channels through mechanisms such as “regulatory volume decrease” (RVD) and “regulatory volume increase” (RVI) to restore osmotic balance. It is worth noting that plants such as Salicornia europaea also exhibit similar salinity stress responses, achieving salinity tolerance by altering cell structure and aquaporin expression. This suggests that similar osmoregulatory principles may be widely present across different biological kingdoms [7].

Nevertheless, current research on salinity-driven volume changes, osmotic responses, and the underlying molecular basis of *Penaeus monodon* hemocytes remains limited. Most available studies emphasize whole-animal physiology or tissue-level ion regulation, whereas integrative analyses linking quantitative hemocyte volume phenotypes to transcriptome-wide programs and gene-network architecture are still scarce. Moreover, how hemocyte volume plasticity is coordinated with the regulation of ion transporters, aquaporins, stress signaling, and ribosome-centered gene networks has not been systematically examined. Here, hemocyte volume dynamics and morphology were quantified in vitro across hypoosmotic, isosmotic, and hyperosmotic salinity conditions and complemented with osmotic regulation assays using permeant (urea) and non-permeant (sorbitol) solutes. RNA sequencing, differential expression and KEGG enrichment analyses, weighted gene co-expression network analysis (WGCNA), and protein–protein interaction (PPI) network construction were integrated to identify salinity- and volume-associated modules and hub genes, followed by qRT-PCR validation of representative osmoregulatory and stress markers. In addition, because osmotic perturbation can be accompanied by oxidative imbalance through mitochondrial metabolic remodeling and endoplasmic reticulum proteostasis stress, ROS levels and antioxidant indices (SOD, POD, CAT, and T-AOC) were quantified, and redox- and antioxidant-related pathways were examined within the enrichment and network analyses to strengthen antioxidant-oriented mechanistic interpretation. Collectively, this work establishes an integrative cellular and molecular framework for hemocyte osmoregulation and provides a basis for mechanistic dissection of salinity tolerance in euryhaline crustaceans.

## 2. Materials and Methods

### 2.1. Experimental Animals and Rearing Conditions

The experimental *P. monodon* were purchased from the Shenzhen Base of the South China Sea Fisheries Research Institute, Chinese Academy of Fishery Sciences, with an average individual weight of 18.31 ± 0.3 g. The experimental seawater had a salinity of 30 ppt. During the rearing process, the water temperature was maintained at 28 ± 1 °C, the pH value at 8.0 ± 0.2, and the dissolved oxygen concentration was not lower than 6 mg/L. The experimental tank was a recirculating water system equipped with a sand filter and a protein separator. The shrimp were fed twice a day with compound feed, with the feeding amount being 3–5% of the body weight. Before the experiment, 10 individuals were randomly selected for microscopic observation and routine PCR detection to confirm the absence of significant pathogen infection, ensuring the reliability and reproducibility of the experimental results.

### 2.2. Experimental Design Overview

Two in vitro experimental series were performed to distinguish ionic (salinity/NaCl) effects from pure osmotic effects on hemocyte responses. (i) In the salinity-stress experiment, L15-based media were adjusted to salinities 0–50 by NaCl addition. (ii) In the osmotic-control experiments, hemocytes were maintained in the basal L-15 medium background (“0 ppt” nominal label; without additional NaCl) and challenged with exogenous solutes, sorbitol (impermeant) or urea (permeant), to vary osmotic pressure without altering the NaCl-based salinity gradient ionic composition.

### 2.3. Hemolymph Collection and Hemocyte Preparation

Hemolymph was collected from the dorsal sinus of the third abdominal segment using a sterile syringe and immediately mixed 1:1 (*v*/*v*) with anticoagulant solution (450 mM NaCl, 10 mM KCl, 10 mM EDTA, 10 mM HEPES, pH 7.4). The mixture was gently inverted and centrifuged at 4 °C and 800× *g* for 10 min to pellet hemocytes. After removing the supernatant, hemocytes were resuspended in L15 basic culture medium (isotonic 30 ppt unless otherwise specified) for subsequent experiments.

### 2.4. Salinity Stress Experiment Design

To evaluate NaCl-driven salinity (ionic) effects on hemocytes, L-15-based media were adjusted to nominal salinities 0–50 ppt by NaCl addition only, and hemocytes prepared as described in Section 2.3 were incubated in each salinity condition for 24 h. Each salinity culture medium was prepared using Leibovitz’s L-15 medium (1×) as the basal medium (Sigma-Aldrich, St. Louis, MO, USA). The standard L-15 formulation contains inorganic salts including NaCl (8.0 g·L^−1^), KCl (0.4 g·L^−1^), CaCl_2_ (0.1396 g·L^−1^), MgCl_2_ (0.0937 g·L^−1^), MgSO_4_ (0.0977 g·L^−1^), KH_2_PO_4_ (0.06 g·L^−1^), and Na_2_HPO_4_ (0.19 g·L^−1^), with other components (amino acids, vitamins, galactose/pyruvate, etc.) provided as in the commercial formulation. “0 ppt” denotes the NaCl-addition 0 condition (L-15 base medium without additional NaCl), and its measured osmolality (306.67 ± 4.99 mOsm·kg^−1^) mainly reflects the background ionic/solute components of L-15 rather than a salt-free condition. For the salinity series (0, 5, 10, 20, 30, 40, and 50 ppt), media were generated by supplementing L-15 with NaCl only and calibrating with an osmometer (OSMOMAT 3000 osmometer (Gonotec GmbH, Berlin, Germany); Table 1). For reproducibility, the corresponding added NaCl-equivalent concentrations (calculated from Δosmolality relative to 0 ppt assuming ~2 mOsm·kg^−1^ per 1 mM NaCl) were approximately: 5 ppt, +36.83 mM (+2.15 g·L^−1^); 10 ppt, +138.00 mM (+8.06 g·L^−1^); 20 ppt, +273.17 mM (+15.96 g·L^−1^); 30 ppt, +420.66 mM (+24.58 g·L^−1^); 40 ppt, +560.67 mM (+32.77 g·L^−1^); and 50 ppt, +589.50 mM (+34.45 g·L^−1^). When combined with the basal NaCl in L-15 (8.0 g·L^−1^; ~136.89 mM), the estimated total NaCl concentrations were ~173.72, 274.89, 410.06, 557.56, 697.56, and 726.39 mM for Salinity 5–50 ppt, respectively. Importantly, no other ions were adjusted; thus, “salinity” is used as a nominal label rather than a full seawater-salt ionic composition, and hypo-/hyperosmotic interpretations should be based on the measured osmolality values in Table 1. To improve interpretability of the experimental design, we used a two-tier strategy. First, hemocyte morphology and volume were quantified across a broader NaCl-adjusted L-15 salinity series (5, 10, 20, 30, 40, and 50 ppt) after 24 h incubation to map the overall volume–osmolality response pattern. Second, downstream assays that are more resource-intensive (oxidative stress/antioxidant indices and RNA-seq) were performed on three representative salinities (5 ppt, 30 ppt, and 50 ppt), selected to capture a low–reference–high contrast based on measured osmolality and to align with the reference rearing salinity (30 ppt) while bracketing the strongest physiological divergence observed across the gradient. All endpoints were collected at the same 24 h time point to enable cross-assay integration under a standardized exposure duration. Each salinity treatment was set up with three independent biological replicates (from different individuals). For morphological/volume distribution statistics, at least 200 cells were randomly counted per replicate. For molecular detection (RNA extraction, qRT-PCR/transcriptome), cell pellets were collected separately for each replicate and stored at −80 °C. All operations were carried out under sterile conditions, with all other culture conditions being consistent except for salinity to exclude interference from non-salinity factors.

### 2.5. Osmotic Regulation Experiment

In the osmolyte (sorbitol/urea) experiments, the reference (baseline) medium refers to the basal L-15 medium background (0 ppt; 306.67 ± 4.99 mOsm·kg^−1^; Table 1), rather than the NaCl salinity-gradient 30 ppt condition (1148.0 ± 9.8 mOsm·kg^−1^; Table 1). Sorbitol or urea was added to the basal L-15 medium to generate the indicated osmolyte series, and the osmolality of each condition was individually measured and calibrated using an osmometer (Table 2). Because these osmolyte series were conducted in a substantially lower osmolality window (~350–450 mOsm·kg^−1^; Table 2) than the NaCl 30 ppt condition, absolute cell diameters between the two experimental paradigms are not intended to be compared directly; interpretations are based on within-series comparisons.

### 2.6. Hemocyte Separation and Morphological Measurement

Hemocytes were prepared as described in Section 2.3. The cells were resuspended in the corresponding treatment medium and dropped onto a slide for observation with a laser confocal microscope (Leica/Stellaris 5). Ten random fields of view were photographed for each group, and the single-cell diameter was measured using ImageJ software (Version 1.54p, 17 February 2025; National Institutes of Health, Bethesda, MD, USA) (at least 30 samples per group), with the volume calculated according to the spherical volume formula V = 4/3πr^3^. In addition, to assess cell membrane integrity, the cell viability was detected by 0.4% trypan blue staining. The proportion of stained and unstained cells was counted to ensure that the cell activity was maintained above 90% during the experiment.

Vesicle quantification and counting. Cytoplasmic vesicles were quantified from the same microscopic images used for morphological measurement. For each treatment, vesicles were counted in at least 30 randomly selected intact hemocytes collected from 10 random fields of view. In ImageJ, each cell boundary was manually outlined as the region of interest (ROI), and vesicles were scored within the cytoplasmic ROI (excluding the nucleus). A “vesicle” was defined as a distinct, round/oval, phase-lucent vacuole-like structure with a clear boundary inside the cytoplasm (as indicated by red arrows in Supplementary Figure S1C). Small dense puncta lacking a clear lumen (granules), irregular edge blebs at the cell perimeter, and extracellular debris were not counted. Vesicle number was reported as vesicles per cell (mean ± SD).

### 2.7. Osmotic Pressure Determination Method

The osmotic pressure of different salinity, urea, and sorbitol treatment solutions was measured using the freezing point depression method. After preparation, each experimental solution was left to stand for 24 h to ensure that the solute was fully dissolved and reached a stable state. The osmotic pressure was measured using a micro-osmometer. 100 μL of the sample was injected into the instrument measurement pool, measured three times repeatedly, and the average value was taken as the final osmotic pressure value of the treatment group. The instrument was calibrated with a standard solution (290 mOsm·kg^−1^) before each measurement to ensure the accuracy of the readings. The osmotic pressure of the salinity series solutions (0, 5, 10, 20, 30, 40 and 50 ppt) ranged from approximately 300 to 1500 mOsm·kg^−1^, while urea and sorbitol treatments produced narrower ranges (Table 1). Notably, “0 ppt” corresponds to the L15 base medium without NaCl supplementation, and its osmolality represents the basal-medium background rather than a 0 mOsm·kg^−1^ condition. For the urea treatment (0–50 mM), osmotic pressure values were in an intermediate range of approximately 400–1000 mOsm·kg^−1^, whereas the sorbitol treatment (0–50 mM) produced more moderate increases, from about 350 to 450 mOsm·kg^−1^ (Table 2). All measurements were carried out under constant temperature conditions (25 ± 1 °C). After measurement, the samples were immediately discarded, and the instrument measurement head was rinsed three times with deionized water to prevent solute residue.

### 2.8. Reactive Oxygen Species (ROS) and Antioxidant Indices

After 24 h incubation under the designated salinity treatments, hemocytes from each biological replicate were collected by centrifugation at 4 °C, gently washed once with ice-cold phosphate-buffered saline (PBS, pH 7.4) to remove residual medium, and immediately lysed on ice in RIPA lysis buffer (Beyotime Biotechnology, Shanghai, China; P0013B). Lysates were clarified by centrifugation at 4 °C, and the supernatants were used for subsequent assays. Total protein concentration was determined using a BCA protein assay kit (Thermo Fisher Scientific, Waltham, MA, USA; 23225) according to the manufacturer’s instructions. Reactive oxygen species (ROS) level was determined using a commercial ELISA kit (Reactive Oxygen Species (ROS) ELISA Kit; cat. no. ml026288; Shanghai Enzyme-linked Biotechnology Co., Ltd., Shanghai, China) according to the manufacturer’s protocol. Briefly, standards and hemocyte samples (hemocyte lysates; clarified by centrifugation and the supernatant was used for measurement) were added to the antibody-precoated microplate and incubated with the biotin-labeled detection antibody. After washing, streptavidin–HRP was added, followed by TMB substrate for color development. The reaction was stopped and absorbance was read at 450 nm using a microplate reader. Activities of superoxide dismutase (SOD), peroxidase (POD), and catalase (CAT), as well as total antioxidant capacity (T-AOC), were measured in the same hemocyte lysates using commercial assay kits (Nanjing Jiancheng Bioengineering Institute, Nanjing, China) following the manufacturers’ protocols. All ROS and antioxidant indices were normalized to total protein content for downstream statistical analyses, consistent with the biological replicate design used throughout the study.

### 2.9. RNA Extraction and Transcriptome Sequencing

Hemocytes were collected from the 5, 30, and 50 ppt groups of the salinity experiment, and total RNA was extracted using TRIzol reagent (Invitrogen, Carlsbad, CA, USA). The purity of RNA was detected by NanoDrop 2000 (Thermo Fisher Scientific, Wilmington, DE, USA) (A260/A280 between 1.9–2.1), and the integrity was assessed by Agilent 2100 Bioanalyzer (Agilent Technologies, Santa Clara, CA, USA) (RIN ≥ 8.0). Library construction was performed using the NEBNext Ultra RNA Library Prep Kit (New England Biolabs, Ipswich, MA, USA), and sequencing was carried out on the Illumina NovaSeq 6000 platform (Illumina, San Diego, CA, USA). The raw reads obtained were processed by the fastp software (v0.19.5) to remove adapter sequences and low-quality data, and then aligned to the reference genome using HISAT2 (v2.2.1). Across libraries, sequencing generated 40.23–45.77 million clean reads per sample with Q30 = 95.35–96.25%, and 53.72–75.89% of reads were mapped to the reference genome (Table S2). Gene expression levels were quantified as gene-level raw read counts for differential expression testing, and FPKM values were calculated and used only for visualization and expression display. Differential expression analysis was performed using DESeq2 (v1.50.2) based on the raw count matrix, whereas FPKM-based values were used only for plotting/heatmap visualization. Gene-level raw counts were obtained by counting reads mapped to each annotated gene from the aligned RNA-seq data, and the resulting count matrix was used as input for DESeq2. Sequencing services were provided by Meige Biotechnology Co., Ltd. (Guangzhou, China).

### 2.10. Enrichment Analysis and Network Construction

Differentially expressed genes were subjected to GO functional classification and KEGG metabolic pathway enrichment analysis to identify the main biological processes under salinity stress. For WGCNA, module–trait associations were performed using sample-level traits that were available for all RNA-seq samples (*n* = 9; 5 ppt, 30 ppt, and 50 ppt, three biological replicates each). Because replicate-matched continuous cell-volume values were not available for the RNA-seq samples, cell volume was not used as a WGCNA trait. Instead, salinity treatment was encoded as dummy (0/1) indicator variables (5 ppt, 30 ppt, and 50 ppt) and used for module–trait correlation analysis. Measured medium osmolality values corresponding to each treatment (Table 1) were included as an auxiliary continuous trait for interpretability. Differentially expressed genes from the key module were imported into the STRING database to construct protein–protein interaction (PPI) networks. PPI networks were inferred based on the annotation of the closely related shrimp species *Litopenaeus vannamei*, with a high confidence score (interaction score ≥ 0.7), and the resulting networks were visualized and further analyzed in Cytoscape 3.9 to screen for key regulatory genes.

Gene Set Enrichment Analysis (GSEA) was performed as a pre-ranked analysis implemented in the R package “clusterProfiler” (v4.18.4). For each salinity comparison (5 ppt vs. 30 ppt; 50 ppt vs. 30 ppt), all expressed genes were ranked by the signed log2 fold change (log2FC) from the DESeq2 results (from high to low), and enrichment was tested against KEGG pathway gene sets (map IDs; e.g., MAP00030/MAP00052). The enrichment score (ES) and normalized enrichment score (NES) were computed, and significance was evaluated using gene-set permutation as implemented by the underlying fgsea algorithm in clusterProfiler. Nominal *p*-values were reported together with Benjamini–Hochberg adjusted *p*-values (FDR q-values; reported as *p*adjust). For transparency, both nominal *p*-values and FDR q-values are provided for each reported pathway.

Given the limited sample size in this study (3 salinity groups × 3 biological replicates), WGCNA was used as an exploratory analysis to generate network hypotheses rather than definitive modules. To evaluate robustness, the network construction was repeated under a range of key parameters (soft-threshold power = 6–12, minModuleSize = 20–40, mergeCutHeight = 0.20–0.30), and module assignment consistency was summarized by module overlap. In addition, a leave-one-out (LOO) sensitivity analysis was performed by re-running WGCNA after removing one sample at a time to assess whether the major module–trait associations and top-ranked intramodular genes were stable.

### 2.11. qRT-PCR Validation

Typical genes such as Na^+^/K^+^-*ATPase*, AQP4, HSP70, and CHOP were selected for quantitative PCR validation. RNA reverse transcription was performed using the PrimeScript RT Kit (Takara Bio, Kusatsu, Japan), and real-time fluorescent quantitative PCR was completed on the ABI 7500 instrument. The reaction system had a total volume of 20 μL, and the cycling conditions were 95 °C pre-denaturation for 30 s, followed by 40 cycles (95 °C for 5 s, 60 °C for 30 s). β-actin and EF-1α were used as internal reference genes, and the relative expression levels were calculated using the 2^−ΔΔCt^ method. Reference-gene stability under salinity/osmotic stress was evaluated by examining Ct distributions across the 24 h salinity treatments (5, 30, and 50 ppt). For *β-actin* (ACTIN), Ct values showed low variability (5 ppt: 17.61 ± 0.23; 30 ppt: 17.43 ± 0.25; 50 ppt: 17.32 ± 0.29 cycles) and did not differ significantly among salinity groups (one-way ANOVA: F(2,6) = 0.939, *p* = 0.442; Levene’s test *p* = 0.951). For *EF-1α* (EF), Ct values were similarly stable across treatments (5 ppt: 18.42 ± 0.27; 30 ppt: 18.93 ± 0.21; 50 ppt: 18.56 ± 0.29 cycles), with no significant group differences (one-way ANOVA: F(2,6) = 0.917, *p* = 0.375; Levene’s test *p* = 0.932). Therefore, *β-actin* and *EF-1α* were considered sufficiently stable for normalization, and target-gene expression was normalized to the geometric mean of these two reference genes for the 2^−ΔΔCt^ calculation. All samples were set up with three technical and biological replicates. The primer sequences are provided in Table S1.

### 2.12. Data Processing and Statistical Analysis

The unit of replication (n) refers to independent biological replicates rather than individual cells. For cell-based measurements (e.g., hemocyte diameter/volume and vesicle number), multiple hemocytes were quantified within each biological replicate, and the per-replicate mean value was used for statistical inference to avoid pseudo-replication. For biochemical assays (ROS and antioxidant indices), each biological replicate represents an independently prepared hemocyte sample derived from an independent pool prepared from different shrimp and processed independently.

Assumptions were evaluated on biological-replicate values. Normality was assessed using the Shapiro–Wilk test, and homogeneity of variances was assessed using the Brown–Forsythe (or Levene) test. When assumptions were met, one-way ANOVA followed by Tukey’s multiple comparisons was applied; when variance heterogeneity was detected, Welch’s ANOVA followed by Dunnett T3 multiple comparisons was used. Given the small n for some biochemical endpoints (n = 3), nonparametric Kruskal–Wallis tests with Dunn’s multiple comparisons were additionally used as a sensitivity check. Exact *p*-values (two-sided) for omnibus tests and key post hoc comparisons are reported in Section 3 and figure legends.

## 3. Results

### 3.1. Volume and Morphological Changes in P. monodon Hemocytes Under Different Salinities and Exogenous Osmotic Pressures

**Figure 1:**
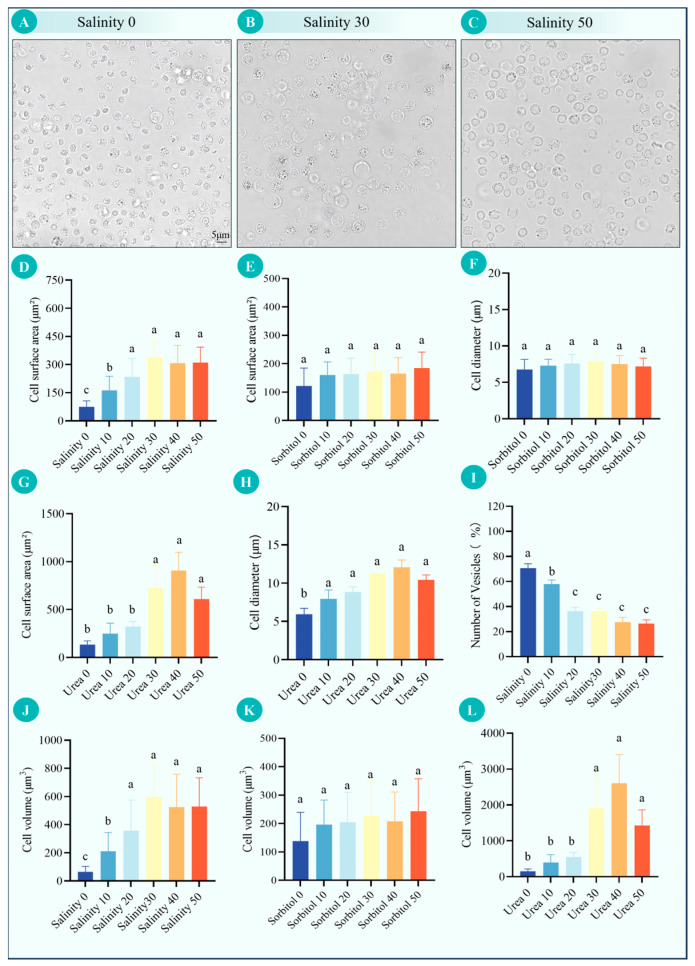
Morphological and volumetric changes in hemocytes of *P. monodon* under different salinities and exogenous osmotic pressures. (**A**–**C**) Representative micrographs of hemocytes under 0 ppt, 30 ppt, and 50 ppt, respectively, showing the differences in cell volume and morphology at different salinities. (**D**) Changes in the average cell volume of hemocytes under different salinity treatments. (**E**,**F**) Changes in cell diameter and volume in the sorbitol treatment groups. (**G**,**H**) Changes in cell diameter and volume in the urea treatment groups. (**I**) Statistical Analysis of Vesicle Number in Hemocytes in Response to Salinity Stress. (**J**–**L**) Cell volume (μm^3^) under salinity (**J**), sorbitol (**K**), and urea (**L**) treatments, respectively. Vesicles were quantified per cell according to the criteria described in Section 2.6. Data are presented as mean ± SD. Different lowercase letters indicate significant differences among treatments for the same endpoint/parameter (one-way ANOVA followed by Tukey’s post hoc test, *p* < 0.05). Bars sharing the same letter are not significantly different.

Significant volume and morphological changes were observed in *P. monodon* hemocytes after 24 h incubation under different salinities (Figure 1A–D,J). At 0 ppt (L15 base medium without added NaCl; 306.67 ± 4.99 mOsm·kg^−1^), cells appeared smaller and slightly contracted, with an average diameter of 6.88 ± 2.33 μm and a volume of 73.6 ± 20.60 μm^3^. As salinity increased, cell volume gradually expanded. At 10 ppt, the volume was 151.20 ± 66.90 μm^3^, and at 20 ppt, it reached 220.40 ± 100.10 μm^3^. The maximum volume was observed at 30 ppt, where cells had an average diameter of 13.09 ± 1.13 μm and a volume of 322.40 ± 110.70 μm^3^. Microscopic observations revealed that cells were significantly swollen, with increased membrane tension, clear cell edges, and evenly distributed cytoplasm. Further increases in 40 ppt and 50 ppt resulted in slight volume reductions to 311.70 ± 96.50 μm^3^ and 300.30 ± 93.50 μm^3^, respectively, with average diameters of 12.67 ± 1.02 μm and 12.74 ± 1.14 μm. At these salinities, cell morphology remained stable without rupture or deformation. Consistently, 0.4% trypan blue staining indicated that hemocyte viability remained >90% across treatments after 24 h incubation.

Although low salinity is typically expected to trigger an immediate osmotic swelling and high salinity to cause rapid shrinkage, the volumes reported here were measured after 24 h and therefore reflect steady-state regulation rather than the initial water-influx/efflux phase. The smaller/contracted morphology observed at 0 ppt may indicate a rapid RVD process following an early swelling episode (net solute efflux and cytoskeletal compaction), and/or a shift in hemocyte sub-populations (e.g., preferential loss/reduced representation of larger, more hydrated cells) under the most hypotonic condition. In contrast, the maximal swelling around 30 ppt suggests that this condition is closer to a physiologically permissive osmolality window for maintaining hemocyte hydration in vitro. Moreover, because this salinity series was generated by supplementing L15 with NaCl only (rather than reconstituting a full seawater-like ionic composition), ionic-composition effects may also contribute to the non-monotonic volume pattern beyond total osmolality. Consistent with this interpretation, hemocyte volume increased continuously from 0 ppt to 30 ppt and then showed a modest downward trend at higher salinities (40 and 50), while cell morphology remained stable and membranes remained intact without obvious rupture or deformation.

In the sorbitol osmolyte experiment, hemocyte volume exhibited only a mild increase across exogenous solute concentrations (Figure 1F,G,K,L and Figure S1A). Under 0, 10, 20, 30, 40, and 50 mM sorbitol conditions, average volumes were 122.03 ± 62.87 μm^3^, 160.51 ± 45.83 μm^3^, 163.61 ± 56.23 μm^3^, 173.51 ± 68.80 μm^3^, 165.50 ± 56.46 μm^3^, and 184.96 ± 56.13 μm^3^, respectively. The volume increase fluctuated slightly with concentration but remained relatively small. According to osmotic pressure measurements, sorbitol treatment only produced a moderate increase in external osmolality, from approximately 350 to 450 mOsm·kg^−1^ (Table 2), which was much smaller than the osmotic differences induced by the salinity treatments, and the corresponding changes in hemocyte volume were also milder. Cell diameters ranged from 7.29 ± 0.88 μm to 7.88 ± 1.29 μm, with no significant differences observed (*p* > 0.05). Notably, the osmolyte (sorbitol/urea) experiments were performed over a markedly lower osmolality range (~350–450 mOsm·kg^−1^; Table 2) than the NaCl salinity-gradient 30 ppt condition (~1148 mOsm·kg^−1^; Table 1). Therefore, the absolute hemocyte diameters reported in the osmolyte series are not intended to be directly compared with those from the NaCl 30 ppt condition; the key interpretation relies on within-series differences across osmolyte levels. Microscopic observations showed that cells in all treatment groups were round and intact, with smooth membrane surfaces, no obvious shrinkage or uneven swelling, uniform cell distribution, and no cell debris formation.

In contrast, although the urea solutions showed higher measured osmolality than the sorbitol solutions (Table 2), urea is membrane-permeant and therefore does not sustain extracellular effective hypertonicity in the same way as non-permeant sorbitol. Accordingly, the urea series is interpreted as eliciting a more transient transmembrane osmotic gradient that can equilibrate as urea diffuses, while also altering intracellular osmolyte balance and macromolecular interactions, rather than imposing a stronger maintained extracellular hypertonic challenge per se. At the Urea 40 condition, hemocytes had the largest volume, with an average diameter of 12.32 ± 0.85 μm. As the concentration increased to 50 mM, cell volume slightly decreased. Microscopic observations revealed that cells in the urea treatment group were significantly swollen, with plump cytoplasm and clear membrane structures. Some high-concentration samples showed uneven cell sizes and increased individual variation. At Urea 50, a few cells exhibited reduced volume or localized collapse, but overall cell morphology remained intact without rupture.

### 3.2. Oxidative Stress and Antioxidant Responses of Hemocytes Under Representative Salinity Conditions

**Figure 2:**
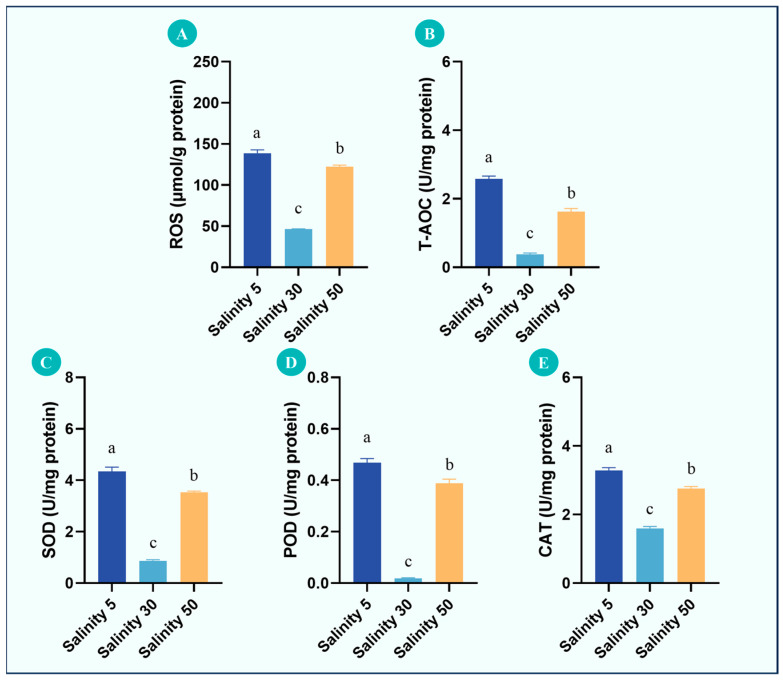
Hemocytes were incubated for 24 h at 5 ppt, 30 ppt, and 50 ppt. (**A**) Reactive oxygen species (ROS) content in hemocytes. (**B**) Total antioxidant capacity (T-AOC) in hemocytes. (**C**) Superoxide dismutase (SOD) activity in hemocytes. (**D**) Peroxidase (POD) activity in hemocytes. (**E**) Catalase (CAT) activity in hemocytes. Data are presented as mean ± SD (n = 3). n indicates independent biological replicates (not individual cells). Statistical procedures, assumption checks, and exact *p*-values for omnibus and key post hoc comparisons are reported in Section 2.12 and the corresponding Results text. Statistical significance was determined by one-way ANOVA followed by Tukey’s post hoc test. Different lowercase letters indicate significant differences among treatments for the same endpoint/parameter (one-way ANOVA followed by Tukey’s post hoc test, *p* < 0.05). Bars sharing the same letter are not significantly different.

To assess redox status under osmotic challenge, ROS content and antioxidant indices were quantified in hemocytes after 24 h incubation at representative low salinity (5 ppt), reference salinity (30 ppt), and high salinity (50 ppt) (Figure 2A–E). ROS levels were substantially higher at 5 ppt (138.85 ± 3.98 μmol/g protein) than at 30 ppt (46.29 ± 0.50 μmol/g protein) and remained elevated at 50 ppt (122.18 ± 2.14 μmol/g protein) relative to 30 ppt (Figure 2A). Consistently, total antioxidant capacity (T-AOC) increased markedly at 5 ppt (2.58 ± 0.08 U/mg protein) and 50 ppt (1.62 ± 0.09 U/mg protein) compared with 30 ppt (0.38 ± 0.04 U/mg protein) (Figure 2B).

Antioxidant enzyme activities showed a similar salinity-dependent pattern. SOD activity was higher at 5 ppt (4.34 ± 0.17 U/mg protein) and 50 ppt (3.54 ± 0.04 U/mg protein) than at 30 ppt (0.87 ± 0.04 U/mg protein) (Figure 2C). POD activity increased at 5 ppt (0.469 ± 0.015 U/mg protein) and 50 ppt (0.389 ± 0.016 U/mg protein) relative to 30 ppt (0.0187 ± 0.0025 U/mg protein) (Figure 2D). CAT activity also increased at 5 ppt (3.28 ± 0.09 U/mg protein) and 50 ppt (2.76 ± 0.06 U/mg protein) compared with 30 ppt (1.60 ± 0.06 U/mg protein) (Figure 2E). Overall, both low- and high-salinity exposures were associated with increased ROS levels accompanied by coordinated activation of antioxidant capacity and enzyme activities in hemocytes relative to the reference salinity condition. In terms of biological magnitude (effect size), relative to the reference 30 ppt condition, 5 ppt and 50 ppt increased ROS by ~3.00-fold (Δ = +92.56 μmol/g protein) and ~2.64-fold (Δ = +75.89 μmol/g protein), respectively; T-AOC by ~6.77-fold (Δ = +2.20 U/mg protein) and ~4.26-fold (Δ = +1.24 U/mg protein); SOD by ~5.00-fold (Δ = +3.47 U/mg protein) and ~4.08-fold (Δ = +2.67 U/mg protein); POD by ~25.05-fold (Δ = +0.450 U/mg protein) and ~20.77-fold (Δ = +0.370 U/mg protein); and CAT by ~2.06-fold (Δ = +1.69 U/mg protein) and ~1.73-fold (Δ = +1.16 U/mg protein).

Statistical reporting for Figure 2 endpoints (n = 3 biological replicates per group). Shapiro–Wilk tests did not indicate clear departures from normality within groups (all *p* > 0.27), and Brown–Forsythe tests did not detect significant variance heterogeneity for these endpoints (ROS *p* = 0.353834; T-AOC *p* = 0.614492; SOD *p* = 0.249606; POD *p* = 0.494250; CAT *p* = 0.865167). One-way ANOVA showed significant treatment effects for all five indices: ROS F(2,6) = 1059.294, *p* = 2.252317 × 10^−8^; T-AOC F(2,6) = 659.521, *p* = 9.284621 × 10^−8^; SOD F(2,6) = 908.310, *p* = 3.567514 × 10^−8^; POD F(2,6) = 1042.308, *p* = 2.363882 × 10^−8^; CAT F(2,6) = 475.192, *p* = 2.469204 × 10^−7^. As a robustness check under potential variance heterogeneity and small n, Welch’s ANOVA remained significant for all endpoints (all *p* ≤ 5.205276 × 10^−5^), and Kruskal–Wallis tests also supported overall group differences (H(2) = 7.200, *p* = 0.027324). For key pairwise contrasts (Holm-adjusted Welch *t*-tests), 30 ppt differed from both 5 ppt and 50 ppt for all indices (ROS: S5 vs. S30 *p* = 0.001035; S30 vs. S50 *p* = 0.000403; T-AOC: *p* = 0.000166 and 0.000543; SOD: *p* = 0.000798 and 6.62 × 10^−7^; POD: *p* = 0.000854 and 0.000927; CAT: *p* = 6.48 × 10^−5^ and 5.26 × 10^−5^).

### 3.3. Transcriptomic Differences and Functional Enrichment Analysis of P. monodon Hemocytes Under Different Salinity Treatments

**Figure 3:**
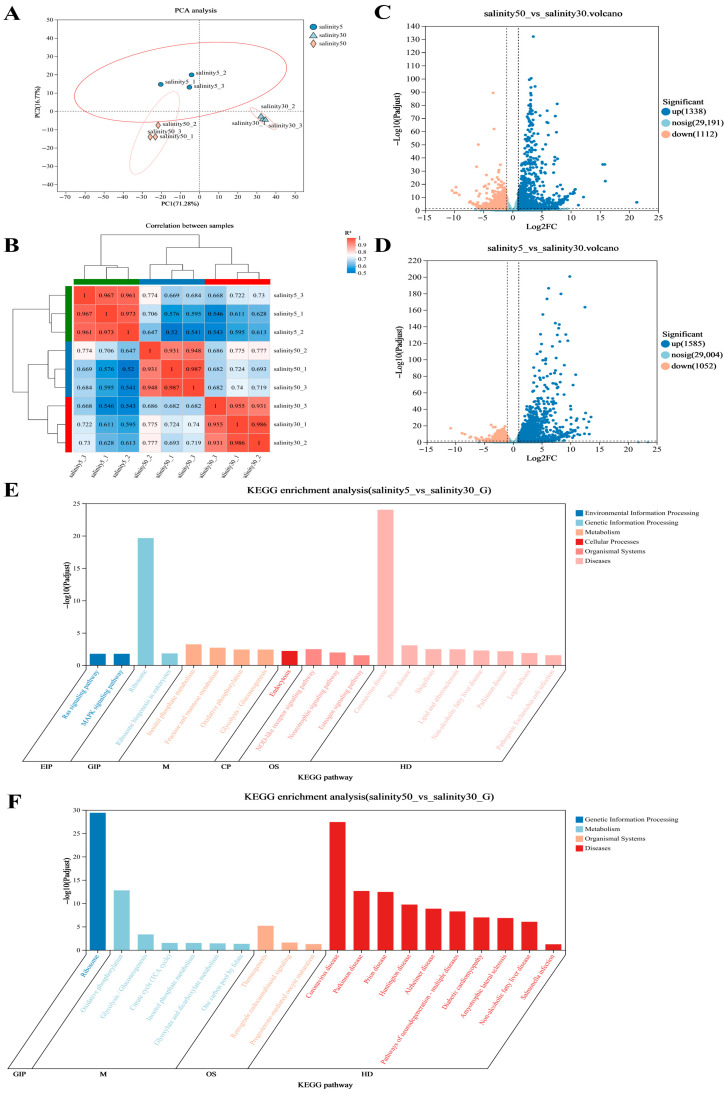
Transcriptomic analysis of hemocytes in *P. monodon* under different salinity treatments. (**A**) Principal component analysis (PCA) plot showing the distribution and clustering of hemocyte samples under 5 ppt, 30 ppt, and 50 ppt conditions. (**B**) Heatmap of inter-sample correlations, displaying expression correlations among samples from different salinity groups. (**C**,**D**) Volcano plots illustrating the distribution of differentially expressed genes (DEGs) in 5 ppt vs. 30 ppt and 50 ppt vs. 30 ppt comparisons. (**E**,**F**) Bar charts of KEGG pathway enrichment analysis, showing significantly enriched functional pathway categories in different salinity comparisons.

RNA-seq analysis yielded high-quality transcriptomic data from hemocyte samples under 5 ppt, 30 ppt, and 50 ppt conditions, with 40.23–45.77 million clean reads per library, Q30 = 95.35–96.25%, and Error rate = 0.0119–0.0124%; overall mapping rates ranged from 53.72–75.89% (uniquely mapped: 33.35–67.16%) (Table S2). Principal component analysis (PCA) results showed clear separation between different salinity groups (Figure 3A). Samples from 5 ppt, 30 ppt, and 50 ppt formed distinct clusters along the first principal component (PC1, 17.28%) and the second principal component (PC2, 7.77%), with good clustering and high reproducibility within groups, indicating that salinity differences significantly impacted gene expression.

The sample correlation heatmap (Figure 3B) revealed that the internal correlation coefficients (R^2^) within each group were all above 0.95, showing good consistency within groups. In contrast, the correlations between different salinity groups were significantly lower, with R^2^ ranging from 0.53–0.74 between 5 ppt and 30 ppt groups, and 0.62–0.77 between 50 ppt and 30 ppt groups, indicating that different salinity treatments caused marked transcriptional expression differences.

Differential expression analysis results are shown in Figure 3C,D. Using the thresholds of |log_2_FC| ≥ 1 and FDR (*p*adj) < 0.05, a large number of differentially expressed genes (DEGs) were identified in both the 5 ppt vs. 30 ppt and 50 ppt vs. 30 ppt comparisons. The substantial repertoire of DEGs indicates that changes in salinity led to extensive transcriptional reprogramming in hemocytes. Volcano plots showed that DEGs were widely distributed across the full expression range, with a subset of genes exhibiting large expression changes (|log_2_FC| > 10) and highly significant FDR values.

KEGG enrichment analysis results (Figure 3E,F) indicated that in the comparison between 5 ppt and 30 ppt, significantly enriched pathways included MAPK signaling pathway, Ribosome, Protein processing in endoplasmic reticulum, Oxidative phosphorylation, Glycolysis/Gluconeogenesis, and Carbon metabolism. In the comparison between 50 ppt and 30 ppt, significantly enriched pathways included Protein processing in endoplasmic reticulum, Ribosome, Apoptosis, MAPK signaling pathway, Aminoacyl-tRNA biosynthesis, and Antigen processing and presentation. Some metabolism-related pathways (such as Glycolysis, TCA cycle) and signal transduction pathways (such as Calcium signaling pathway, cAMP signaling pathway) also showed varying degrees of enrichment.

### 3.4. Gene Co-Expression Modules and Trait Association Analysis Under Different Salinities in P. monodon Hemocytes

**Figure 4:**
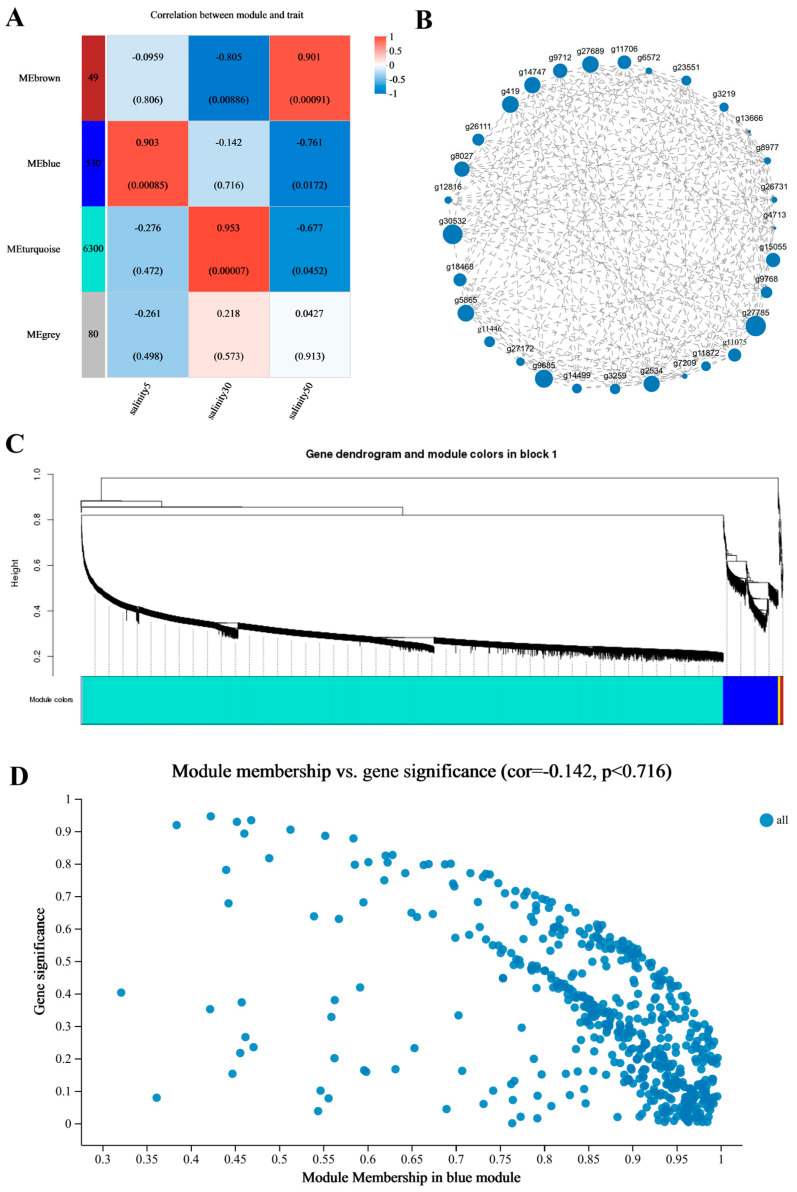
Weighted gene co-expression network analysis (WGCNA) of hemocytes in *P. monodon* under different salinity conditions. (**A**) Heatmap of module–trait (salinity) relationships, showing the correlation coefficients and significance levels between different co-expression modules and 5 ppt, 30 ppt, and 50 ppt conditions. (**B**) Gene co-expression network of the Blue module, with node size representing the connectivity of genes within the module. (**C**) Gene clustering dendrogram and module assignment results, with different colors representing distinct co-expression modules. (**D**) Scatter plot of module membership versus gene significance, displaying the correlation distribution among genes in the Blue module.

Based on the transcriptomic data of hemocytes under different salinity conditions, a co-expression network was constructed using Weighted Gene Co-expression Network Analysis (WGCNA), and several gene modules significantly related to salinity were identified (Figure 4A–D). A total of four main modules were detected, namely the brown, blue, turquoise, and grey modules, containing 49, 530, 6300, and 80 genes, respectively.

The correlation analysis between modules and phenotypic traits (5 ppt, 30 ppt, 50 ppt) showed that the brown module was significantly positively correlated with 50 ppt (r = 0.901, *p* = 0.00091), the blue module with 5 ppt (r = 0.903, *p* = 0.00085), the turquoise module with 30 ppt (r = 0.953, *p* = 0.00007), while the grey module did not show significant correlation. The significant differences between modules under different salinities indicated that genes in each module had distinct expression patterns.

Module–trait relationships were computed using the trait matrix defined in Methods (Section 2.10), where salinity treatment was represented as dummy (0/1) indicators (5 ppt/30 ppt/50 ppt). Because osmolality values are constant within each treatment group, the module–trait correlations primarily reflect group-level differences among the three salinity conditions rather than within-group continuous variation.

The network structure of the blue module (Figure 4B) showed tight gene connectivity, forming a high-density interaction network. The high correlation between nodes indicated that some genes were located in the central area of the network with high connectivity. Notably, several ribosomal protein–coding genes ranked highly by intramodular connectivity (network centrality), indicating that they are central nodes within the co-expression topology of this module; however, such “hubness” here reflects network structure rather than a definitive trait-driving role. Because ribosomal/translation-related genes frequently emerge as high-degree nodes in co-expression networks due to high expression abundance and coordinated stress-responsive regulation, their network centrality here is interpreted primarily as a generic “translation activity” signature rather than direct evidence of upstream regulatory control. Accordingly, these ribosomal genes are reported as candidate markers for follow-up validation rather than definitive causal regulators.

The gene clustering tree analysis results (Figure 4C) showed that genes were clustered into several independent modules based on their expression patterns, with clear module boundaries and high consistency in gene expression within each module. The turquoise module had the largest number of genes, forming a long branch in the clustering tree, while the blue and brown modules formed separate clustering branches.

Further analysis showed that the correlation between gene significance (GS) and module membership (MM) was weak and not significant (r = −0.142, *p* = 0.716; Figure 4D). This indicates that module membership does not systematically track gene–trait significance in this dataset, and the module–trait linkage should be interpreted cautiously. Accordingly, the reported “hub” genes are presented as network-central candidates for hypothesis generation (high intramodular connectivity and/or high MM) rather than as definitive trait-associated drivers, given the modest sample size.

### 3.5. Protein–Protein Interaction Network and Gene Set Enrichment Analysis Under Different Salinities in P. monodon Hemocytes

**Figure 5:**
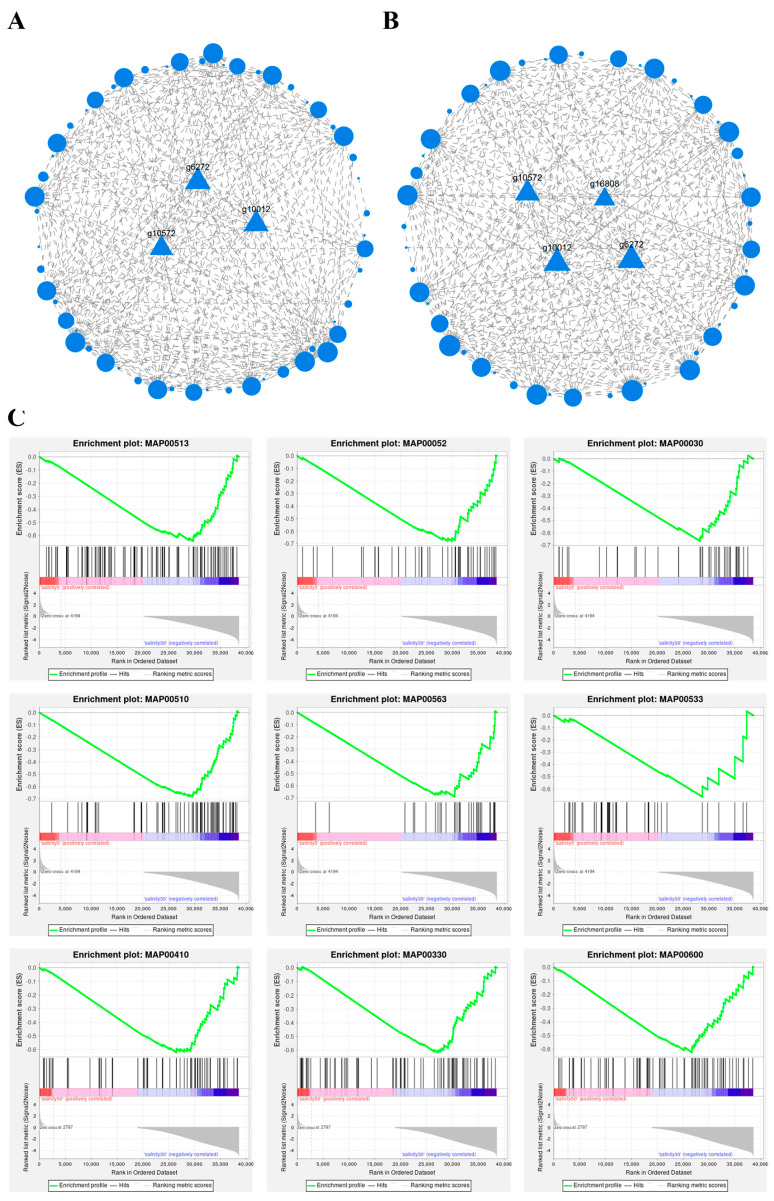
Protein–protein interaction network and gene set enrichment analysis of hemocytes in *P. monodon* under different salinity treatments. (**A**,**B**) Protein–protein interaction (PPI) networks constructed from differentially expressed genes, showing the interaction relationships among genes in 5 ppt vs. 30 ppt and 50 ppt vs. 30 ppt comparisons, respectively. Node size indicates gene connectivity. (**C**) Gene set enrichment analysis (GSEA) results, illustrating significantly enriched metabolic and signaling pathways in different salinity comparisons. In each plot, the green curve represents the enrichment score (ES) trend, vertical lines indicate the positions of genes in the ranked list, and the gray shaded area at the bottom shows the distribution of the ranking metric.

The protein–protein interaction (PPI) network based on differentially expressed genes revealed distinct connectivity patterns in hemocytes under different salinity conditions (Figure 5A,B). Because the PPI network was inferred via ortholog-based mapping to a related species annotation, the displayed interactions and node centrality should be interpreted as predicted/conserved associations rather than species-specific experimentally validated PPIs in *P. monodon*. In the comparison between 5 ppt and 30 ppt, the network had a more dispersed structure, mainly composed of several moderately interacting nodes, with some genes located in the central area of the network. In contrast, the comparison between 50 ppt and 30 ppt showed a more compact network structure with dense node connections, forming several high-association core gene clusters. In both networks, several ribosomal-related genes were detected in central positions, including *RPL40*, *RPS30*, *RPL31*, and *RPL9*, which had high node degree and connectivity, forming stable interaction cores in the network.

Gene Set Enrichment Analysis (GSEA) further identified pathways showing enrichment trends under different salinity treatments (Figure 5C). In the comparison between 5 ppt and 30 ppt, the top enriched gene sets included “MAP00513—various types of N-glycan biosynthesis”, “MAP00052—galactose metabolism”, “MAP00030—pentose phosphate pathway”, “MAP00510—N-glycan biosynthesis”, “MAP00563—GPI-anchor biosynthesis”, “MAP00533—glycosaminoglycan biosynthesis”, “MAP00410—beta-alanine metabolism”, “MAP00330—arginine and proline metabolism”, and “MAP00600—sphingolipid metabolism.” The ES values of these gene sets were negative, indicating that the leading-edge genes tended to be shifted toward the lower end of the pre-ranked list in 5 ppt vs. 30 ppt. Nominal *p*-values for these pathways were <0.05, whereas the Benjamini–Hochberg adjusted FDR q-values (*p*adjust) did not reach conventional significance thresholds; therefore, these GSEA results are interpreted as enrichment trends rather than definitive statistically significant discoveries. Notably, the negative enrichment of the pentose phosphate pathway (MAP00030), a major NADPH-generating route, is compatible with a salinity-dependent shift in cellular reducing-equivalent supply that is relevant to antioxidant systems. In parallel, enrichment signals involving oxidative phosphorylation, together with pathways linked to protein processing in the endoplasmic reticulum and MAPK signaling (Figure 5C), suggest coordinated remodeling of mitochondrial energy metabolism and proteostasis-associated stress programs across salinity conditions.

There was partial gene overlap between different enriched pathways, mainly involving protein processing, sugar metabolism, and amino acid metabolism. The enrichment curves showed consistent enrichment trends for each pathway, with core genes concentrated at the negative end of the sorted list. Combined with the PPI network, it was evident that the above ribosomal genes had potential associations with multiple metabolic pathways.

### 3.6. Relative Expression Changes in Key Genes Under Different Treatments in P. monodon Hemocytes

**Figure 6:**
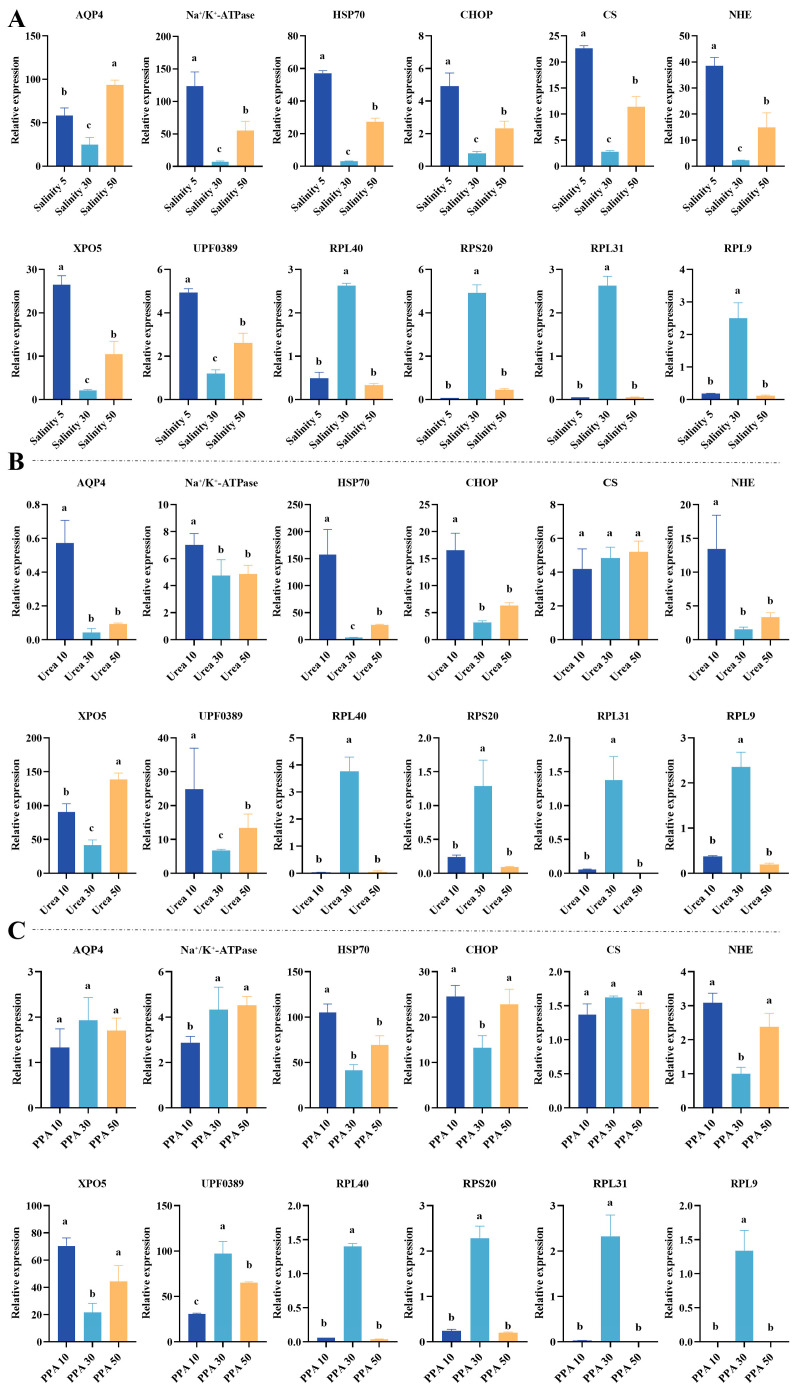
Analysis of relative expression levels of key genes in hemocytes of *P. monodon* under different salinity and exogenous osmotic pressure treatments. (**A**) Relative expression levels of osmoregulation- and stress-related genes (*AQP4*, Na^+^/K^+^-*ATPase*, *HSP70*, *CHOP*) and ribosomal protein genes (*RPL40*, *RPS20*, *RPL31*, *RPL9*) under different salinity conditions (5 ppt, 30 ppt, 50 ppt). (**B**) Relative expression levels of the aforementioned genes under different urea treatments. (**C**) Relative expression levels of the genes under different sorbitol treatments. Bar graphs represent mean ± SD. Significant differences were determined by one-way analysis of variance (ANOVA). Different lowercase letters (a–c) above the bars indicate significant differences among treatments for the same gene (one-way ANOVA followed by Tukey’s multiple-comparisons test, *p* < 0.05). Bars sharing the same letter are not significantly different.

qRT-PCR results showed that the expression of several osmoregulatory and ribosomal-related genes in *P. monodon* hemocytes changed significantly under different osmotic pressure treatments, including salinity, urea, and sorbitol (Figure 6). Under salinity treatment, the expression levels of *AQP4*, Na^+^/K^+^-*ATPase*, *HSP70*, and *CHOP* showed significant differences. The average expression levels of *AQP4* under 5 ppt, 30 ppt, and 50 ppt were 58.20 ± 8.83, 25.00 ± 7.88, and 93.64 ± 5.55, respectively. Na^+^/K^+^-*ATPase* had average expression levels of 123.59 ± 21.89, 7.32 ± 1.16, and 55.02 ± 14.32, respectively. *HSP70* had average expression levels of 57.10 ± 1.60, 3.03 ± 0.18, and 27.36 ± 2.13, while *CHOP* had average expression levels of 4.91 ± 0.82, 0.80 ± 0.11, and 2.33 ± 0.44. Overall, *AQP4* and *HSP70* increased under high salinity, while Na^+^/K^+^-*ATPase* and *CHOP* significantly decreased under moderate salinity.

Ribosomal-related genes *RPL40*, *RPS20*, *RPL31*, and *RPL9* also responded significantly to salinity changes. The average expression levels of *RPL40* under 5 ppt, 30 ppt, and 50 ppt were 0.493 ± 0.136, 2.629 ± 0.055, and 0.330 ± 0.041, respectively. *RPS20* had average expression levels of 0.078 ± 0.004, 4.930 ± 0.365, and 0.451 ± 0.049, respectively. *RPL31* had average expression levels of 0.049 ± 0.003, 2.627 ± 0.222, and 0.057 ± 0.004, while *RPL9* had average expression levels of 0.183 ± 0.012, 2.507 ± 0.472, and 0.116 ± 0.025. The increases in *RPS20* and *RPL31* were the most significant, with approximately 63-fold and 54-fold increases under 30 ppt compared to the 5 ppt group.

Under urea treatment, the gene response trends were different. The expression level of *AQP4* was 0.573 ± 0.12 at 0 mM urea, decreased to 0.043 ± 0.017 at 30 mM urea, and slightly increased to 0.095 ± 0.003 at 50 mM urea. Na^+^/K^+^-*ATPase* and *HSP70* both reached the highest levels at 0 mM urea (HSP70: 157.36 ± 37.50) and then gradually decreased with increasing urea concentration. *CHOP* had the highest expression at low urea concentration (16.54 ± 2.10) and significantly decreased at medium and high concentrations. Among ribosomal-related genes, *RPL40* and *RPL9* increased at 30 mM urea (3.77 ± 0.49 and 2.36 ± 0.27, respectively) but decreased to the lowest levels at 50 mM urea (<0.1 on average). *RPL31* and *RPS20* maintained low expression levels under urea treatment. Under sorbitol treatment, the expression changes in each gene were generally small. *AQP4*, Na^+^/K^+^-*ATPase*, *HSP70*, and *CHOP* showed slight fluctuations between different concentration treatments.

All qRT-PCR fold changes were normalized to the geometric mean of *β-actin* and *EF-1α*; both reference genes showed stable Ct values across salinity treatments (ACTIN: ANOVA *p* = 0.442; EF: ANOVA *p* = 0.375), supporting the reliability of normalization under salinity stress.

## 4. Discussion

### 4.1. Volume and Morphological Responses of P. monodon Hemocytes Under Different Salinities and Exogenous Osmotic Pressures

The volume changes in hemocytes are important indicators of osmoregulatory capacity and can directly reflect the cellular adaptation to changes in external osmotic pressure. In this study, the trend of *P. monodon* hemocyte volume first increasing and then decreasing under different salinities indicates a significant volume regulatory capability [8]. Within the low to medium salinity range (0–30 ppt), hemocyte volume significantly expanded with increasing external osmotic pressure, showing the cellular response characteristics of active water absorption or increased internal osmotic pressure. This volume increase may be related to the accumulation of soluble osmoregulatory substances within the cell (such as free amino acids, potassium ions, glycine betaine, etc.), which maintain homeostasis by regulating the osmotic potential difference between the inside and outside of the cell. When salinity further increased to 40–50, the slight decrease in cell volume suggested that the cells had completed the initial osmotic balance and initiated the regulatory volume decrease (RVD) response, achieving cellular homeostasis by expelling ions or organic osmolytes. This reversible volume regulation is considered a key process for crustaceans to maintain cellular integrity in environments with fluctuating salinity [9].

The non-monotonic volume pattern observed across the salinity series should be interpreted in the context of the assay time scale. Because hemocyte size was quantified after 24 h exposure, the reported volumes represent a short-term regulated steady state shaped by RVD/RVI processes and potentially by changes in hemocyte sub-population composition, rather than the immediate osmotic swelling/shrinkage response occurring within minutes. Therefore, the smaller mean volume at 0 ppt is compatible with sustained RVD and/or population shifts after an early swelling phase, while the larger mean volume around 30 ppt may reflect a more permissive osmotic window for maintaining hemocyte hydration in vitro. A limited time-course sampling (e.g., 0.5–2 h, 6 h, and 24 h) would directly test whether an early canonical swelling phase is followed by RVD/RVI-driven compensation and/or hemocyte sub-population shifts that shape the apparent 24 h mean volume pattern.

At higher salinities, the slight reduction in hemocyte volume without overt rupture indicates that the cell membrane has considerable mechanical stability and osmotic resilience. Under these hyperosmotic conditions, cells appear to maintain structural integrity through controlled volume contraction, suggesting the synergistic action of the membrane cytoskeleton and ion regulatory systems. Membrane channel proteins such as *AQP4* and Na^+^/K^+^-*ATPase* are likely to play key roles at this stage by regulating transmembrane water and ion fluxes to prevent excessive cell swelling or lysis.

Further insights into the sources of different osmotic effects were revealed by exogenous solute treatment. Sorbitol, an impermeable solute, mainly causes an increase in extracellular osmotic pressure without directly affecting intracellular ionic balance [10]. In this study, sorbitol treatment only caused mild volume fluctuations, indicating that hemocytes can maintain volume stability through extracellular-intracellular ion flux in a purely hypertonic environment, demonstrating passive osmoresistance. In contrast, urea is a permeable solute that can freely diffuse into the cell and change the intracellular osmotic potential in a short time. The results showed that urea treatment caused significant volume expansion, especially peaking at 30–40 mM, suggesting an osmotic equilibration lag effect in cells under the influence of permeable solutes. The rapid diffusion of urea into the cell caused a temporary mismatch in osmotic equilibrium between the inside and outside of the cell, causing the cell to temporarily absorb water and swell [11].

It is worth noting that the comparison between urea and sorbitol treatment indicates that the response of *P. monodon* hemocytes to osmotic pressure changes is not only dependent on the external solute concentration but also closely related to the solute membrane permeability. The dramatic volume changes caused by urea reflect the high responsiveness of the cell membrane to permeable osmolytes, while the mild effect of sorbitol indicates that the cells can maintain high stability in a non-permeable hypertonic environment. This difference reveals that hemocytes in natural salinity fluctuations have both rapid volume regulation capabilities and the ability to achieve mechanical stability through membrane channels and cytoskeleton regulation, a complex adaptive mechanism.

Mechanistically, measured osmolality reflects total solute osmolality of the medium, but the effective osmotic stress across the plasma membrane depends strongly on solute permeability (effective tonicity). Sorbitol is largely non-permeant over the experimental timescale and therefore maintains extracellular hypertonicity, promoting water efflux and volume reduction followed by regulatory volume increase (RVI). In contrast, urea is membrane-permeant and can diffuse into cells, which reduces the persistence of the extracellular-to-intracellular osmotic gradient; thus, urea may drive a different volume trajectory that reflects rapid osmotic equilibration, intracellular osmolyte redistribution, and potential impacts on protein stability/macromolecular interactions rather than a stronger maintained extracellular hypertonic exposure.

The present work focused on acute hemocyte responses to salinity shifts over a 24 h time scale. Although this exposure duration does not capture the full spectrum of chronic acclimation processes that occur over days to weeks, it is relevant to short-term salinity perturbations commonly experienced by farmed and wild shrimp, such as heavy rainfall, rapid freshwater inflow, or evaporation-driven salinity spikes in shallow ponds. Importantly, microscopic examination in this study confirmed that hemocyte membrane integrity and overall morphology were preserved across the 0–50 ppt treatments after 24 h, indicating that the observed transcriptomic changes primarily reflect active cellular regulation rather than nonspecific effects of extensive cell death. A further limitation is inherent to the ex vivo hemocyte culture paradigm. Hemolymph collection followed by anticoagulant exposure and centrifugation/washing (EDTA–NaCl–sodium citrate–based handling) may impose handling-related stress or activation that is not present in vivo. In addition, hemocytes were maintained under serum-free L-15 conditions, which lack native hemolymph proteins, hormones, and immune factors that can modulate osmotic and redox responses. Finally, the salinity media were generated by NaCl-only supplementation of L-15 without adjusting other ions; thus, the ionic composition may deviate from hemolymph and seawater-equivalent salt mixtures, and interpretations should be anchored to measured osmolality rather than assuming full in vivo ionic realism. Nevertheless, longer-term in vivo experiments integrating survival, growth and performance metrics under controlled salinity regimes will be required to fully connect these acute cellular responses to chronic salinity tolerance at the whole-animal level.

### 4.2. Redox Regulation Under Osmotic Stress

The results from this study suggest that hemocytes of *Penaeus monodon* actively regulate redox balance in response to osmotic stress. The increased ROS levels observed in both low (5 ppt) and high (50 ppt) salinity conditions were paralleled by a significant activation of antioxidant defenses, as reflected by the enhanced activities of SOD, POD, and CAT, as well as increased T-AOC. These findings indicate that oxidative stress is an important component of hemocyte response to osmotic perturbations and that antioxidant systems are crucial for protecting cells from the damaging effects of ROS [12].

At low salinity (5 ppt), hemocytes experienced a substantial increase in ROS levels, which was accompanied by a more pronounced activation of antioxidant enzymes. This suggests that hypoosmotic stress, which can transiently alter cell hydration and increase membrane tension while perturbing ion fluxes, leads to a significant oxidative challenge. Notably, microscopic examination and trypan blue staining indicated preserved morphology and >90% viability after 24 h across salinity treatments, arguing against overt membrane rupture under our in vitro conditions; therefore, the oxidative response is interpreted as a regulated stress adaptation rather than a nonspecific consequence of membrane damage. In contrast, at high salinity (50 ppt), ROS levels were elevated to a lesser extent, indicating that hyperosmotic stress primarily affects ionic balance and dehydration. However, despite the difference in the nature of the stressors, both conditions resulted in activation of antioxidant responses, highlighting the critical role of redox regulation in hemocytes’ ability to cope with osmotic stress.

The strong correlation between ROS production and antioxidant enzyme activities suggests that hemocytes are well-equipped to manage oxidative stress through coordinated activation of antioxidant pathways. The upregulation of SOD, POD, and CAT indicates that these enzymes play essential roles in mitigating oxidative damage caused by changes in osmotic pressure. The increase in T-AOC further supports this view, reflecting a broader antioxidant response designed to counteract the oxidative burden imposed by osmotic stress [13]. A methodological limitation is that ROS in this study was quantified using an ELISA kit, which provides a kit-defined ROS-related oxidative status proxy rather than direct measurement of short-lived intracellular ROS species. Thus, the ROS result is interpreted as reflecting relative differences in ROS-related oxidative status across treatments, not species-resolved ROS dynamics or real-time ROS flux. Future work could strengthen mechanistic inference by incorporating orthogonal assays (e.g., fluorescent probes for intracellular ROS, lipid peroxidation such as MDA/4-HNE, protein carbonyls, and/or 8-OHdG) to validate oxidative-stress conclusions beyond a single proxy endpoint.

These findings underscore the importance of redox regulation in cellular osmoregulation and highlight the need for further investigations into the molecular mechanisms underlying redox signaling in hemocytes under osmotic stress conditions.

### 4.3. Transcriptomic Differences and Functional Enrichment Analysis of P. monodon Hemocytes Under Different Salinity Treatments

The transcriptomic analysis results show that salinity changes induce significant genome-wide expression reconstruction in hemocytes, reflecting the systemic regulatory pattern of *P. monodon* in response to osmotic stress. PCA and sample correlation analysis show that samples under different salinities are clearly clustered and separated, indicating that salinity is the main environmental factor driving transcriptional differences in hemocytes. This clear expression stratification not only reflects the direct impact of external osmotic pressure but also implies that hemocytes activate specific physiological adaptation programs under different salinity environments.

From the number and magnitude of differentially expressed genes (DEGs), the salinity response process involves extensive molecular participation. A large number of DEGs were detected in the comparisons between 5 ppt and 30 ppt and between 50 ppt and 30 ppt, indicating significant transcriptional reprogramming of hemocytes in both low-salinity and high-salinity environments. This bidirectional regulatory trend suggests that hemocytes need to not only deal with cell swelling and ion loss in hypotonic environments but also prevent dehydration and ion accumulation in hypertonic environments, thereby regulating membrane transport, energy metabolism, and protein homeostasis maintenance in different directions.

Functional enrichment analysis reveals that salinity changes mainly affect three major categories of biological processes: protein processing, energy metabolism, and signal transduction. Among them, the significant enrichment of the MAPK signaling pathway and Protein processing in the endoplasmic reticulum indicates that salinity stress activates typical cellular stress signaling pathways. The MAPK pathway is considered an important bridge mediating environmental signals and transcriptional regulation in crustacean osmoregulation and can regulate the expression of stress genes such as *HSP70* and *CHOP* through phosphorylation cascades, thereby maintaining intracellular protein homeostasis and anti-apoptotic balance. The significant enrichment of the endoplasmic reticulum protein processing pathway further indicates that under salinity fluctuations, hemocytes enhance protein folding and repair mechanisms to cope with proteotoxic stress caused by osmotic stress [14]. It should be noted that these pathway-level conclusions are derived from RNA-seq enrichment/network analyses, whereas the current qRT-PCR validation focuses on selected transport/stress genes and ribosomal genes; additional validation using 2–3 representative targets from MAPK/ER processing/oxidative phosphorylation will further strengthen the pathway interpretation in future work.

In addition, the high significant enrichment of the Ribosome pathway is a key finding of this study. Ribosomal genes show strong responses in both low-salinity and high-salinity conditions, indicating that the protein synthesis system plays a core role in osmotic stress adaptation. Studies have shown that changes in osmotic pressure can regulate the rate of ribosome biogenesis through the mTOR and eIF signaling axes, thereby adjusting translational activity to adapt to cellular energy status and synthetic needs. The large-scale coordinated expression of ribosomal-related genes in *P. monodon* hemocytes may reflect the cell rebalancing of protein synthesis and energy consumption under different salinities. Combined with subsequent WGCNA analysis, it can be seen that genes such as *RPL40*, *RPS30*, *RPL31*, and *RPL9* have higher connectivity in low-salinity environments, which is consistent with a coordinated translation-activity/translation reprogramming signature; however, network connectivity alone does not establish upstream regulatory roles, and these genes are therefore treated as candidate markers pending functional validation [15].

The differential enrichment of metabolic pathways also reveals the reshaping of energy utilization strategies under salinity stress. The activity of pathways such as Glycolysis, TCA cycle, and Oxidative phosphorylation indicates that hemocytes accelerate energy metabolism to support ion pump activity and osmoregulatory responses when coping with salinity changes; while the enrichment of amino acid metabolism pathways (such as glycine, serine, and alanine metabolism) may be related to the accumulation of osmotic organic matter, providing osmoprotection for cell volume regulation.

### 4.4. Gene Co-Expression Modules and Trait Association Analysis Under Different Salinities in P. monodon Hemocytes

The WGCNA analysis revealed that the gene expression in *P. monodon* hemocytes under different salinity conditions exhibited highly modular characteristics, indicating that the transcriptional regulatory network has distinct organization and synergy. The modular expression structure reflects the division of labor and collaboration of different functional genes in salinity response, providing a systematic perspective to understand the complex osmoregulation process.

A key limitation is the modest sample size for co-expression network inference (n = 9), which may reduce module stability and the reliability of hub prioritization. Although we performed sensitivity analyses (parameter sweeps and/or leave-one-out re-fitting), these results should be interpreted cautiously and primarily as exploratory hypotheses. Future work with larger independent cohorts and orthogonal validation (e.g., qPCR across additional biological replicates, perturbation assays, or validation against external transcriptomic datasets when available) will be required to confirm module reproducibility and the functional relevance of candidate hub genes.

The significant correlation between modules and salinity traits indicates that hemocytes activate specific functional modules under different salinities rather than random gene responses. The blue module is highly correlated with low-salinity conditions (5 ppt), while the brown module is positively correlated with high-salinity conditions (50 ppt), suggesting that cells adopt different molecular strategies in low-salinity and high-salinity environments. Low-salinity environments typically lead to cell swelling and increased pressure of ion leakage, so the genes in the blue module may mainly participate in volume regulation, ion absorption, and osmotic homeostasis maintenance. In contrast, high-salinity environments cause dehydration and ion influx, and the enrichment of the brown module indicates that cells activate stress protection and metabolic compensation mechanisms under these conditions. The turquoise module is significantly correlated with medium-salinity conditions, representing a transitional expression state that may correspond to a relatively stable physiological level of the cells [16].

From a network structure perspective, the high connectivity and strong aggregation within the blue module suggest that it forms a stable co-expression cluster in osmotic response. Nodes with high connectivity are often treated as putative hub genes in co-expression networks; however, given the modest sample size here, these hubs should be regarded as candidates for prioritization rather than definitive regulators of the trait. The blue module is rich in ribosomal structural protein genes (such as *RPL40*, *RPS30*, *RPL31*, *RPL9*), suggesting a prominent translational reprogramming signature under low-salinity conditions. indicating that the protein synthesis system is centrally regulated under low-salinity conditions. The coordinated expression of these ribosomal genes is usually associated with systematic adjustments in translation rates, which may reflect the cell response to energy and protein homeostasis demands under low-salinity stress by enhancing or reshaping translational activity [17]. Accordingly, we avoid causal language and describe these ribosomal genes as candidate markers unless functional validation experiments support a regulatory role.

Moreover, the differences between modules also reveal the multi-level nature of salinity response. The tight network and high expression consistency of the blue module indicate strong coupling between the genes in this module, which may act synergistically in the cell osmoregulation process. The significant positive correlation of the brown module with high-salinity conditions suggests enhanced roles in cellular defense and stress response. High-salinity environments can perturb cellular homeostasis and protein conformation, so the genes in this module are likely to participate in protein repair and metabolic energy redistribution processes [18].

The complementary relationship between modules indicates that the salinity adaptation of hemocytes is a dynamic network balance process: volume and translation maintenance under low-salinity conditions, and stress defense and energy metabolism adjustment under high-salinity conditions. Module division not only reveals the synergistic patterns between genes but also provides a basis for subsequent identification of key node genes and their regulatory pathways. Hemocytes are central components of the crustacean immune and homeostatic systems, circulating throughout the hemolymph and directly sensing changes in internal osmotic and ionic conditions. As such, hemocyte-level osmoregulatory and stress responses are expected to be tightly linked to whole-animal performance under fluctuating salinity, by influencing immune competence, metabolic allocation and the capacity to maintain hemolymph homeostasis. The integrative network architecture described here—connecting ion transporters, aquaporins, stress chaperones and ribosomal hubs—therefore provides a mechanistic cellular framework that can help to interpret previously reported salinity-dependent patterns of growth, survival and disease susceptibility in *P. monodon* and related penaeid shrimps.

### 4.5. Protein–Protein Interaction Network and Key Gene Expression Regulation Mechanisms Under Different Salinities in P. monodon Hemocytes

The protein–protein interaction (PPI) network based on differentially expressed genes reveals that the gene regulation in *P. monodon* hemocytes under different salinity conditions presents a highly organized interaction pattern. Compared with medium salinity (30 ppt), the network topology differences under low-salinity and high-salinity conditions are significant, indicating that salinity changes not only cause changes in the expression levels of individual genes but also reshape the association structure between genes. The relatively dispersed network structure under low-salinity conditions suggests that cells may be in the initial stage of stress response, with multiple regulatory modules not yet forming stable connections. In contrast, the significant enhancement of node connections and increased network centralization under high-salinity conditions indicate that cells activate a tighter response network in a sustained hypertonic environment to maintain metabolic and protein homeostasis.

In different networks, ribosomal-related genes are consistently found in central positions, especially genes such as *RPL40*, *RPS30*, *RPL31*, and *RPL9*, which show high node degree and connectivity. These patterns indicate that ribosomal genes are associated with the salinity response of hemocytes and tend to occupy central positions in the co-expression and PPI networks at the transcriptomic level. The ribosome is not only the main structural unit for protein synthesis but also an integration hub for environmental response signals, whose composition and function can dynamically adjust according to environmental conditions. The central location of ribosomal protein genes in the network suggests that cells may coordinate control over energy allocation, protein processing, and stress repair by regulating the structure and efficiency of the translational apparatus [19].

The GSEA enrichment results indicate that the significant enrichment of sugar metabolism and glycosylation-related pathways under low-salinity conditions, while most metabolic pathways have negative enrichment scores, suggesting that in a hypotonic environment, hemocytes reduce metabolic load by inhibiting energy metabolism and biosynthetic pathways to alleviate the energy pressure caused by osmotic stress. The enrichment of glycosylation and lipid metabolism pathways implies that cell membrane structure may be adjusted to enhance stability against osmotic pressure changes. Under high-salinity conditions, the upregulation of protein processing, ribosome function, and amino acid metabolism pathways reflects the compensatory role of cells in reinforcing the protein synthesis and folding repair system under hypertonic stress. The overall trend shows that the metabolic pathway remodeling induced by salinity changes is a process centered on protein homeostasis regulation [18].

The qRT-PCR validation results further support this inference. The significant upregulation of Na^+^/K^+^-*ATPase* and *AQP4* indicates that ion transport and water channels play key roles in maintaining cellular osmotic balance, while the expression changes in *HSP70* and *CHOP* reflect the involvement of cellular stress signals and endoplasmic reticulum protein homeostasis regulation. Particularly noteworthy is *HSP70*, which, as a molecular chaperone, plays a key role in maintaining protein folding and endoplasmic reticulum protein homeostasis. Under diverse stress conditions, HSP70 can buffer the accumulation of misfolded proteins and help protect intracellular proteins from damage [20]. Therefore, the upregulation of *HSP70* may represent a key molecular bridge that links osmoregulation with protein quality control in *P. monodon* hemocytes [21]. It is also worth noting that the expression of *RPL40*, *RPS20*, *RPL31*, and *RPL9* significantly increases under medium-salinity conditions but decreases under high-salinity conditions, indicating that the activation of the ribosomal system is closely related to the cell stable osmotic state. This trend is consistent with the central position of ribosomal genes in the PPI network, further confirming the key role of the protein synthesis system in the salinity adaptation process [22].

Moreover, the results of urea and sorbitol treatment provide references for distinguishing ionic effects from pure osmotic effects. Urea, which is permeable, can quickly change the intracellular osmotic balance, thereby causing strong fluctuations in ribosomal-related genes and stress genes. Sorbitol, which is impermeable, only forms a hypertonic environment outside the cell and elicits a weaker gene response. This difference indicates that the transcriptional response of hemocytes to salinity changes is not only due to changes in external osmotic pressure but also depends on the degree of ion concentration gradient and the disruption of intracellular homeostasis [23]. The differential response of ribosomal and osmoregulatory genes under different treatments reflects the cell dual perception mechanism for osmotic signals and ionic homeostasis [24].

Overall, *P. monodon* hemocytes form an interaction network in which ribosomal proteins, osmoregulatory channels and stress-response molecules are strongly co-expressed and interconnected under changing salinity conditions, reflecting coordinated regulation at the transcriptomic level. This network not only maintains energy and protein homeostasis in cells under drastic osmotic changes but also provides continuous metabolic support for high-salinity tolerance. This regulatory mode, characterized by the coordination between the translation system and osmoregulatory system, may represent one important molecular layer of the salinity adaptation of *P. monodon* hemocytes, although additional functional studies are required to fully establish its mechanistic role.

## 5. Conclusions

*P. monodon* hemocytes achieve osmotic homeostasis through volume regulation and transcriptional reprogramming in response to salinity changes. Hemocyte volume increased from 0 ppt to 30 ppt and showed a modest decline at 40–50 ppt, while membrane integrity and overall morphology remained preserved and recover volume, demonstrating flexible volume regulatory capabilities and membrane permeability adjustment mechanisms. Transcriptomic and molecular validation results indicate that the MAPK signaling pathway, ribosome biogenesis, endoplasmic reticulum protein processing, and energy metabolism are key pathways in salinity response. *AQP4*, Na^+^/K^+^-*ATPase*, *HSP70*, *CHOP*, and multiple ribosomal protein genes are co-regulated under different salinities, participating in ion transport, stress defense, and protein homeostasis maintenance. Ribosomal-related genes occupy central positions in the co-expression and PPI networks and are closely associated with osmotic adaptation at the gene-expression level. Additionally, ROS production and antioxidant responses play a crucial role in hemocyte adaptation to osmotic stress, with upregulation of SOD, POD, and CAT suggesting effective redox regulation. Further functional work will be needed to verify their causal roles in salinity tolerance. Comprehensive analysis shows that hemocytes effectively maintain structural and functional stability in fluctuating salinity through multi-level integrated regulation of signal transduction, metabolic reconstruction, and protein homeostasis, thereby providing a cellular and molecular basis for the euryhaline adaptation and high salinity tolerance of *P. monodon*.

## Figures and Tables

**Table 1:** Osmolality of NaCl-adjusted Leibovitz’s L-15 media at nominal salinities (0–50 ppt) used in the salinity-stress experiment. (Salinity values 0–50 ppt are nominal labels for NaCl-adjusted L15 media; “0 ppt” = L15 base medium without additional NaCl, and its osmolality reflects the basal ionic/solute components of L15.).

Nominal Salinity	Osmolality (mOsm·kg^−1^)
Salinity 0	306.67 ± 4.99
Salinity 5	380.33 ± 3.09
Salinity 10	582.67 ± 2.05
Salinity 20	853.0 ± 9.09
Salinity 30	1148.0 ± 9.8
Salinity 40	1428.0 ± 17.91
Salinity 50	1485.67 ± 13.6

**Table 2:** Osmolality of Leibovitz’s L-15 media supplemented with urea or sorbitol at the indicated nominal concentrations used in the osmotic-control experiments.

Concentration	Osmolality (mOsm·kg^−1^)	Concentration	Osmolality (mOsm·kg^−1^)
Urea 10	419.0 ± 12.83	Sorbitol 10	348.67 ± 8.96
Urea 20	633.33 ± 18.87	Sorbitol 20	360.67 ± 1.7
Urea 30	717.33 ± 3.4	Sorbitol 30	412.0 ± 1.63
Urea 40	861.0 ± 10.68	Sorbitol 40	434.67 ± 2.05
Urea 50	960.33 ± 15.17	Sorbitol 50	444.67 ± 3.77

## Data Availability

The data presented in this study are openly available in the Genome Sequence Archive (GSA) at the National Genomics Data Center, China National Center for Bioinformation/Beijing Institute of Genomics, Chinese Academy of Sciences (https://ngdc.cncb.ac.cn/gsa) (accessed on 1 December 2025), under accession number GSA: CRA034081.

## Supplementary Materials

The following supporting information can be downloaded at https://www.mdpi.com/article/10.3390/antiox15010147/s1, Figure S1: Morphological and volumetric changes in hemocytes of *P. monodon* under different salinities and exogenous osmotic pressures; Figure S2: Vesicle number changes in hemocytes under sorbitol- and urea-induced osmotic challenges; Table S1: qPCR primer sequences; Table S2: RNA-seq quality-control metrics for each library; Table S3: RNA-seq quality-control metrics for each library; Table S4: Summary of experimental conditions, endpoints, and replication.

## Abbreviations

The following abbreviations are used in this manuscript:

APX1	Ascorbate peroxidase 1
AQP4	Aquaporin 4
CHOP	C/EBP homologous protein
DEGs	Differentially expressed genes
DESeq2	Differential expression analysis for sequence count data
EDTA	Ethylenediaminetetraacetic acid
EF-1α	Elongation factor 1 alpha
FDR	False discovery rate
FPKM	Fragments per kilobase of transcript per million mapped reads
GO	Gene Ontology
GPI	Glycosylphosphatidylinositol
GSEA	Gene Set Enrichment Analysis
HEPES	4-(2-hydroxyethyl)-1-piperazineethanesulfonic acid
HSP70	Heat shock protein 70
KEGG	Kyoto Encyclopedia of Genes and Genomes
MAPK	Mitogen-activated protein kinase
mTOR	Mechanistic target of rapamycin
Na^+^/K^+^-ATPase	Sodium–potassium adenosine triphosphatase
NFAT5	Nuclear factor of activated T cells 5
*P. monodon*	*Penaeus monodon*
PCA	Principal component analysis
PPI	Protein–protein interaction
RNA-seq	RNA sequencing
ROS	Reactive oxygen species
RVD	Regulatory volume decrease
STRING	Search Tool for the Retrieval of Interacting Genes/Proteins
TCA	Tricarboxylic acid cycle
