# Supplementary material for: Integrative Transcriptomic and Network Analysis of Hemocyte Volume Plasticity and Redox Regulation Under Osmotic Stress in Penaeus monodon
Source: Antioxidants (Basel). 2026 Jan 22;15(1):147. doi: 10.3390/antiox15010147 (PMC12838168; doi:10.3390/antiox15010147)
Supplement: Supplementary file 1 [file antioxidants-15-00147-s001.zip › antioxidants-4074096-supplementary.pdf]

Electronic supplementary material

Supplementary Table S1: qPCR primer sequences

Names	Sequences (5'–3')
ACTB	F: GCCCTTGCTCCTTCCACTATC<br>R: CCGGACTCTTCGTACTCATCCT
EF -1α	F: AAGCCAGGTATGGTTGTCAACTTT<br>R: CGTGGTGCATCTCCACAGACT
XPO5	F: ACCTGGGCCTCCAGTTAC<br>R: ACTCACGCTTCATCATCTCC
RPL40	F: TTAGAGGCTATGAGGCTAGCC<br>R: GCAGGGGTGGAGTTGAATT
RPL31	F: TGTGAGACTTTCTCGCCG<br>R: TTACTCAGAGCCTTCATCTACATT
RPL9	F: ACGCTCATTCCAAATGCTGAA<br>R: TACTGCACGCATCTTGTATTGGA
RPS20	F: TTTATACCAGCCTGAAGAAATTGG<br>R: GGACTTGACATTGTTTGAGGTGAG
UPF0389	F: ATGGCGTTGCGAGGAAC<br>R: GTGATCTCTCAGTCCCAATTTTC
CHOP	F: GCCAGGAGGTCAACGTGA<br>R: CTCGCTTTCCTTCCCTGC
CS	F: CTTGCGCGACACCTCC<br>R: TCGGGGAAATAGACGTTTACTGTA
AQP4	F: ATGCTTGTTGCTCGTCATG<br>R: CTCAGGGTCATGCCGA
HSP70	F: TACTCCTGCGTCGGAGTCTT<br>R: ATCGTTTGGCATCAAACACA
Na^+/K^+-ATPase	F: CACCCCACCCAAACAAACT<br>R: TCGTGAACTCTTGCTTTCTTGA
NHE	F: TTGGAGGAGGGGTCTACCTT<br>R: GCTCTCTCCAAAGACAAGCA

Supplementary Table S2: RNA-seq quality-control metrics for each library.

Sample	Clean reads	Clean bases (Gb)	Q30 (%)	Total mapped (%)	Uniquely mapped (%)
salinity5_1	40,233,794	6.014	95.82	67.26	52.89
salinity5_2	43,706,536	6.508	95.79	74.33	63.10
salinity5_3	43,790,032	6.548	95.74	71.38	58.91
salinity30_1	42,480,848	6.332	95.56	75.89	66.99
salinity30_2	41,267,720	6.167	95.51	75.85	67.16
salinity30_3	40,984,918	6.116	95.35	74.80	66.08
salinity50_1	44,191,632	6.600	96.15	54.82	35.02
salinity50_2	45,488,478	6.795	96.22	58.22	39.73
salinity50_3	45,765,444	6.813	96.25	53.72	33.35

Supplementary Table S3: RNA-seq quality-control metrics for each library.

Gene Set Name	Description	Size	ES	NES	Pvalue	Padjust	Rank at MAX
MAP00030	Pentose phosphate pathway	40	-0.66811	-1.19379	0.044266	0.516007	10307
MAP00563	Glycosylphosphatidylinositol (GPI)-anchor biosynthesis	34	-0.69116	-1.21322	0.028254	0.565965	8112
MAP00513	Various types of N-glycan biosynthesis	116	-0.64221	-1.1795	0.004	0.581436	8821
MAP00533	Glycosaminoglycan biosynthesis - keratan sulfate	53	-0.66421	-1.19552	0.03	0.621385	9854
MAP01040	Biosynthesis of unsaturated fatty acids	35	-0.65106	-1.14646	0.122613	0.653481	8019
MAP00220	Arginine biosynthesis	24	-0.64293	-1.10749	0.263425	0.665059	8413
MAP00534	Glycosaminoglycan biosynthesis - heparan sulfate / heparin	22	-0.64372	-1.10317	0.282051	0.673483	10058
MAP00514	Other types of O-glycan biosynthesis	82	-0.6113	-1.11993	0.084	0.689078	13619
MAP00071	Fatty acid degradation	71	-0.63259	-1.14895	0.061061	0.692233	9837
MAP00410	beta-Alanine metabolism	68	-0.60919	-1.1076	0.129	0.701336	10164
MAP00280	Valine, leucine and isoleucine degradation	64	-0.63222	-1.15405	0.059059	0.706383	11885
MAP00860	Porphyrin metabolism	47	-0.62685	-1.12695	0.156	0.710432	9313
MAP00900	Terpenoid backbone biosynthesis	32	-0.63193	-1.10957	0.202811	0.720932	12856
MAP00450	Selenocompound metabolism	15	-0.68289	-1.12035	0.236382	0.732697	9202
MAP00770	Pantothenate and CoA biosynthesis	42	-0.62448	-1.11074	0.178715	0.7511	8863

MAP00510	N-Glycan biosynthesis	76	-0.68282	-1.25077	0.001	0.751897	9020
MAP00061	Fatty acid biosynthesis	21	-0.66606	-1.12762	0.184049	0.762757	7304
MAP00500	Starch and sucrose metabolism	64	-0.62331	-1.13172	0.087	0.77533	8041
MAP00330	Arginine and proline metabolism	86	-0.63188	-1.15462	0.03	0.80042	10126
MAP00052	Galactose metabolism	46	-0.67881	-1.21582	0.029058	0.801213	9421

Supplementary Table S4: Summary of experimental conditions, endpoints, and replication.

Condition	Endpoints measured (24 h)	Replication (n)	Notes
Salinity 0	Hemocyte diameter/volume; morphology	3 biological replicates	≥200 cells counted per replicate for morphology/volume statistics
Salinity 5	Hemocyte diameter/volume; morphology; ROS/T-AOC/SOD/POD/CAT; RNA-seq; qRT-PCR validation	3 biological replicates	ROS/antioxidant endpoints used independent biological replicates (independent hemocyte pools); RNA-seq sampled at Salinity 5/30/50
Salinity 10	Hemocyte diameter/volume; morphology	3 biological replicates	—
Salinity 20	Hemocyte diameter/volume; morphology	3 biological replicates	—
Salinity 30 (reference)	Hemocyte diameter/volume; morphology; ROS/T-AOC/SOD/POD/CAT; RNA-seq; qRT-PCR validation	3 biological replicates	Reference salinity consistent with rearing condition; used as comparator for downstream assays
Salinity 40	Hemocyte diameter/volume; morphology	3 biological replicates	—
Salinity 50	Hemocyte diameter/volume; morphology; ROS/T-AOC/SOD/POD/CAT; RNA-seq; qRT-PCR validation	3 biological replicates	High-salinity contrast; RNA-seq sampled at Salinity 5/30/50

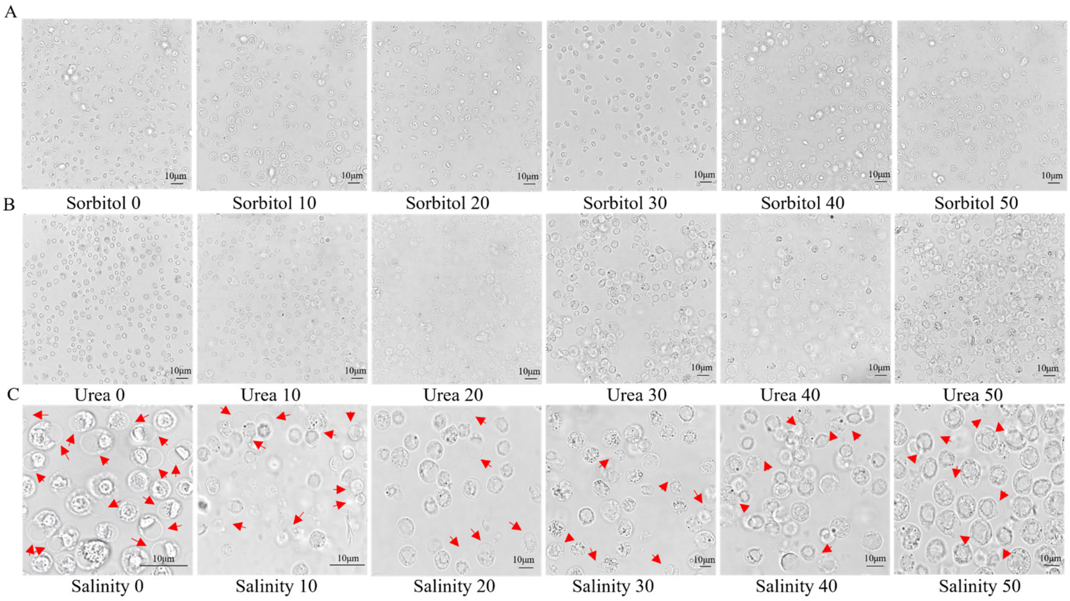

Figure S1 Morphological and volumetric changes in hemocytes of *P. monodon* under different salinities and exogenous osmotic pressures. (A) Micrographs of hemocytes under sorbitol treatment. (B) Micrographs of hemocytes under urea treatment. (C) Micrographs of hemocytes under salinity treatment, with red arrows indicating secretory vesicles. Red arrows indicate cytoplasmic vesicles/vacuoles used for quantification; counting criteria are provided in Section 2.6. Scale bar = 10 μm.

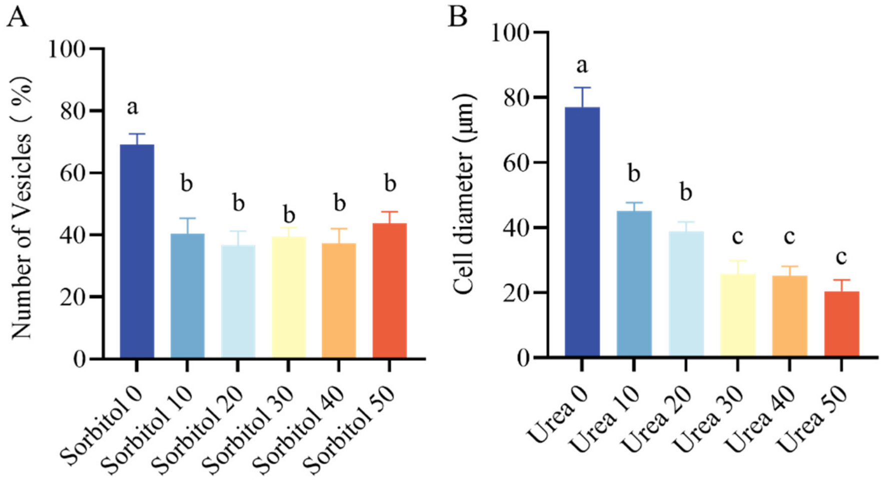

Figure S2. Vesicle number changes in hemocytes under sorbitol- and urea-induced osmotic challenges. (A) Vesicle number (%) in hemocytes under sorbitol treatment at different concentrations. (B) Vesicle number (%) in hemocytes under urea treatment at different concentrations. Different lowercase letters indicate significant differences among treatments within the same panel (one-way ANOVA followed by Tukey's post hoc test, $p < 0.05$). Treatments sharing the same letter are not significantly different.
